# LILRB4 knockdown inhibits aortic dissection development by regulating pyroptosis and the JAK2/STAT3 signaling pathway

**DOI:** 10.1038/s41598-024-66482-3

**Published:** 2024-07-06

**Authors:** Jianxian Xiong, Jiayuan Ling, Jie Yan, Yanyu Duan, Junjian Yu, Wentong Li, Wenbo Yu, Jianfeng Gao, Dilin Xie, Ziyou Liu, Yongzhi Deng, Yongling Liao

**Affiliations:** 1 Department of Cardiovascular Surgery, First Affiliated Hospital of Gannan Medical University, No. 23, Qingnian Road, Zhanggong District, Ganzhou City, 341000 Jiangxi Province China; 2 Heart Medical Centre, First Affiliated Hospital of Gannan Medical University, Ganzhou City, 341000 Jiangxi Province China; 3 Department of Cardiology, First Affiliated Hospital of Gannan Medical University, Ganzhou City, 341000 Jiangxi Province China; 4 Department of Thoracic Surgery, Nankang District First People’s Hospital, Ganzhou City, 341400 Jiangxi Province China; 5 Engineering Research Center of Intelligent Acoustic Signals of Jiangxi Province, Key Laboratory of Prevention and Treatment of Cardiovascular and Cerebrovascular Diseases, Ministry of Education, Gannan Medical University, Ganzhou City, 341000 Jiangxi Province China; 6 Ganzhou Cardiovascular Rare Disease Diagnosis and Treatment Technology Innovation Center, Gannan Medical University, Ganzhou City, 341000 Jiangxi Province China; 7 The First Clinical Medical College, Gannan Medical University, Ganzhou City, 341000 Jiangxi Province China; 8 Department of Cardiovascular Surgery, The Affiliated Hospital of Shanxi Medical University, Shanxi Cardiovascular Hospital (Institute), Shanxi Clinical Medical Research Center for Cardiovascular Disease, Taiyuan, 030024 China

**Keywords:** Aortic dissection, LILRB4, Pyroptosis, The JAK2/STAT3 signaling pathway, Biochemistry, Biological techniques, Genetics, Cardiology

## Abstract

Aortic dissection (AD) is a life-threatening condition with a high mortality rate and without effective pharmacological therapies. Our previous study illustrated that leukocyte immunoglobulin-like receptor B4 (LILRB4) knockdown promoted the contractile phenotypic switch and apoptosis of AD cells. This study aimed to further investigate the role of LILRB4 in animal models of AD and elucidate its underlying molecular mechanisms. Animal models of AD were established using 0.1% beta-aminopropionitrile and angiotensin II and an in vitro model was developed using platelet-derived growth factor BB (PDGF-BB). The effects of LILRB4 knockdown on histopathological changes, pyroptosis, phenotype transition, extracellular matrix (ECM), and Janus kinase 2 (JAK2)/signal transducers and activators of transcription 3 (STAT3) pathways were assessed using a series of in vivo and in vitro assays. The effects of the JAK2 inhibitor AG490 on AD cell function, phenotypic transition, and ECM were explored. LILRB4 was highly expressed in AD and its knockdown increased survival rate, reduced AD incidence, and alleviated histopathological changes in the AD mouse model. Furthermore, LILRB4 knockdown promoted contractile phenotype switch, stabilized the ECM, and inhibited pyroptosis. Mechanistically, LILRB4 knockdown inhibited the JAK2/STAT3 signaling pathway. JAK2 inhibitor AG490 inhibited cell viability and migration, enhanced apoptosis, induced G0/G1 cell cycle arrest, and suppressed S-phase progression in PDGF-BB-stimulated human aortic smooth muscle cells. LILRB4 knockdown suppresses AD development by inhibiting pyroptosis and the JAK2/STAT3 signaling pathway.

## Introduction

Aortic dissection (AD) is a rare, however, life-threatening clinical emergency, particularly when tears accumulate in the aortic arch tissue^1^. Intimal tears in AD trigger inflammatory hyperactivation and extracellular matrix (ECM) degradation, leading to vascular remodeling and aortic wall weakening^2^. Currently, surgery and medication are the primary treatment methods^3^; however, their effectiveness remains unsatisfactory. Therefore, the pathogenic mechanisms of AD must be explored crucially, to identify novel therapeutic targets.

Pyroptosis, also known as secondary necrosis, is a distinct form of non-apoptotic programmed cell death that is closely linked to the inflammatory response^4^. It is driven by proteins from the gasdermin (GSDM) family and involves cell enlargement, lysis, and the release of pro-inflammatory substances, such as ATP, IL-1β, and IL-18^5,6^. Increasing evidence suggests that pyroptosis is involved in AD pathogenesis. In a mouse model of sporadic aortic disease, pyroptosis is triggered by activation of the AIM2 (absent in melanoma 2) inflammasome cascade, which leads to AD and degeneration^7^. High level of adiponectin slows AD progression by inhibiting key regulators in pyroptosis, including gasdermin D (GSDMD), NOD-like receptor family pyrin domain-containing 3 (NLRP3), caspase-1, IL-1β, and IL-18^8^. Additionally, pyroptotic cells are assumed to be more inflammatory and immunogenic than apoptotic cells^9^. A recent study based on bioinformatic analysis revealed that pyroptosis, immune cell infiltration, and their interactions play important roles in AD progression^10^; however, the exact molecular mechanisms remain unclear. Leukocyte immunoglobulin-like receptor B4 (LILRB4) is a member of the leukocyte Ig-like receptor (LILRs) family and is expressed on various immune cells, including B, T, and NK cells. LILRB4 has emerged as a pivotal factor in the treatment of autoimmune disorders, facilitating transplant tolerance induction, and addressing other medical concerns^11^. LILRB4 expression is associated with cardiovascular conditions. LILRB4 deficiency increases the macrophage inflammatory response via NF-κB signaling, which in turn exacerbates atherosclerosis and plaque instability^12^. Moreover, LILRB4 has been observed to affect cardiac hypertrophy development^13,14^. However, reports regarding the involvement of LILRB4 in AD are limited.

The pathogenic Janus kinase (JAK)-signal transducers and activators of transcription 3 (STAT3) signaling pathway can stimulate macrophage effector functions, promote Th17 lymphocyte differentiation, and enhance matrix metalloproteinase expression. These actions ultimately weaken the structural integrity of the vascular walls in inflammation-driven AD^15^. SOCS3, a crucial protein in the JAK/STAT pathway, exerts a beneficial effect in AD when deleted from smooth muscle cells^16^. These findings imply that JAK/STAT signaling is essential for AD development. However, the molecular mechanism by which JAK/STAT modulates AD remains elusive and requires further exploration to establish a robust theoretical framework for clinical utilization.

In our study, we observed that LILRB4 knockdown inhibited AD formation, facilitated contractile phenotype switch, and enhanced ECM stability. In addition, LILRB4 knockdown suppressed pyroptosis and inflammatory responses. Further mechanistic investigation unveiled that the JAK/STAT pathway was significantly inhibited after LILRB4 knockdown. These findings offer novel insights and potential strategies for AD therapy.

## Materials and methods

### Construction of AD mouse model

All animal experiments were approved by The Institutional Animal Care and Use Committee of First Affiliated Hospital of Gannan Medical University. Eighteen C57BL/6 male mice (3 weeks old, weighing 10–12 g) were obtained from SPF Beijing Biotechnology Co., Ltd. (Beijing, China). To construct an animal model of AD, mice were given 0.1% β-aminopropionitrile (BAPN; Sigma-Aldrich, St. Louis, Missouri, USA) in drinking water for four weeks and the body weights were measured and recorded weekly. Angiotensin II (Ang II; 1,000 ng/kg/min; Sigma-Aldrich) was then administered subcutaneously using an Alzet osmotic minipump (model 2004, Durect Corp; Cupertino, CA, USA)^17^. Mice in the control group were administered normal water and then received osmotic minipumps filled with normal saline (0.9% sodium chloride). After 72 h, the mice were deeply anesthetized and euthanized by an intraperitoneal injection of sodium pentobarbital (60 mg/kg). The thoracic aorta of the mice was harvested to observe the formation of AD and perform a pathological examination. In cases of premature death, the mice were promptly dissected and blood accumulation in the thoracic and abdominal cavities was detected to determine whether the aortic coarctation had ruptured. Tissue and blood samples were collected from mice for further analysis. The LILRB4 knockdown model was constructed using adeno-associated virus serotype 2 (AAV2; ViGene Biosciences, Shandong, China), which has broad tropism, allowing it to infect many cell types and tissues, especially the liver. Therefore, AAV2 is a versatile tool in gene therapy and biomedical research^18–20^. All mice were randomly divided into three groups (six per group): control (untreated AD mice), AD + AAV-NC (negative control group), and AD + AAV-shLILRB4 (LILRB4 knockdown group). Mice in the AD + AAV-shLILRB4 group were injected with recombinant AAV-shLILRB4 adenovirus at a titer of 1 × 10^11^ vg/mL in the tail vein. In the AD + AAV-NC group, mice were injected with the same amount of AAV-shNC adenovirus in the same way.

### Cell culture and processing

Human aortic smooth muscle cells (HASMCs) were acquired from icellbioscience biotechnology Co. Ltd. (Shanghai, China). Cells were cultured in SMC medium (B310-500, Sigma-Aldrich) supplemented with 5% fetal bovine serum (Thermo Fisher Scientific, New York, USA), 1% smooth muscle cell growth supplement (311-GS, Sigma-Aldrich), and 100 U/mL penicillin and 100 μg/mL streptomycin in 37 °C with 5% CO_2_. Platelet-derived growth factor BB (PDGF-BB) is an essential mitogen that promotes the phenotypic switching and migration of VSMCs and significantly contributes to AD^21^. To simulate AD in vitro, cells were exposed to PDGF-BB (20 ng/mL; P3201, Sigma-Aldrich) for 12 h. In addition, HASMCs were pre-treated with AG490 (10 μM, Santa Cruz Biotechnology, Santa Cruz, California, USA) for 1 h to inhibit the JAK/STAT signaling pathway.

### Cell transfection

LILRB4 expression in HASMCs was suppressed using siRNAs targeting LILRB4 (si-LILRB4). A non-targeting siRNA (si-NC) was used as a control (Supplementary Table 1 for primer sequences). The cells were seeded into a six-well plate at a density of 1 × 10^5^ cells per well. Once the cell density reached 30–50%, transfection was performed. A total of 20 pmol si-NC or si-LILRB4 was introduced into HASMCs using Lipofectamine 3000 (Thermo Fisher Scientific) following the manufacturer's guidelines.

### Hematoxylin–eosin (HE) staining

At the end of the experiment, the aorta was rapidly removed and cleaned with saline. The entire aorta was fixed in 4% paraformaldehyde for 24 h. Further, aorta tissues were paraffin-embedded and sliced into 4 µm slices. The slices were stained with hematoxylin, rinsed under a running water stream, soaked in 1% hydrochloric acid ethanol, dehydrated, and immersed in an eosin solution. After staining, the sections were sealed with neutral gum and observed under a light microscope (OLYMPUS, Tokyo, Japan).

### Biochemical analysis

Serum samples were collected to assess the levels of alanine aminotransferase (ALT), aspartate aminotransferase (AST), and alkaline phosphatase (ALP) in liver tissues; blood urea nitrogen (BUN) and creatinine (Cr) in kidney tissues; superoxide dismutase (SOD), catalase (CAT), and malondialdehyde (MDA) levels in liver and kidney tissue using a biochemical autoanalyzer (Fuji Medical System, Tokyo, Japan).

### Enzyme-linked immunosorbent assay (ELISA)

Tumor necrosis factor-alpha (TNF-α), interleukin-1 beta (IL-1β), interleukin-8 (IL-8), and interferon-gamma (IFN-γ) levels were measured in serum of mice using ELISA kits (Esebio Biotechnology Co., Ltd., Shanghai, China) according to the manufacturer's instructions.

### Western blot analysis

Protease inhibitor-supplied radioimmunoprecipitation assay (RIPA) buffer (Solarbio, Beijing, China) was used to lyse mouse aortic tissue and HASMCs. Protein samples were separated using 10% SDS-PAGE and electroblotted onto polyvinylidene fluoride (PVDF) membranes (Roche, Basel, Switzerland). The PVDF membranes were coated with 5% defatted milk powder for 2 h. Then PVDF membranes were incubated with primary antibodies (dilution: 1:2000) overnight at 4 °C. The blots were then incubated with the corresponding secondary antibodies for 1 h at room temperature. Protein blots were visualized using ECL luminescent reagent (Amersham, Little Chalfont, UK) and analyzed using the ImageJ software. The primary antibodies in this study included anti-LILRB4 (ab229747, Abcam, Cambridge, UK), anti-α-SMA (ab5694, Abcam), anti-SM22α (ab14106, Abcam), anti-S100A4 (ab197896, Abcam), anti-LOX (ab174316, Abcam), anti-MMP2 (ab250476, Abcam), anti-MMP9 (ab283575, Abcam), anti-NLRP3 (ab263899, Abcam), anti-GSDMD-N (ab215203, Abcam), anti-caspase-1 (D7F10, Cell Signaling, Massachusetts, USA), and anti-β-actin (ab5694, Abcam).

### Cell counting kit-8 (CCK8)

Cells (1 × 10^4^) were seeded in a 96-well plate and cell viability was assessed. Cells were treated with 10 µL of CCK-8 reagent (Solarbio) at predetermined intervals (0, 24, 48, and 72 h) and then cultivated for 2 h. The absorbance was measured at 450 nm using a microplate reader (DALB, Shanghai, China) to quantify cell viability.

### Wound healing assay

The cells were plated in six-well plates at a density of 1 × 10^4^/well. A straight-line scratch was created at a cell concentration of 80% or higher using a pipette tip. Subsequently, cells were grown in serum-free SMCM medium. Images were captured at 0 and 24 h and ImageJ software was used to analyze cell migration.

### Apoptosis and cycle analysis

Apoptosis in HASMCs was measured using FITC-labeled Annexin V and propidium iodide (PI), according to the manufacturer's guidelines. Briefly, a total of 100 µL of cell suspension and 5 µL of FITC-labeled Annexin V were mixed and incubated for 5 min at room temperature in the dark. Subsequently, 5 µL of PI solution was introduced, followed by 400 µL of PBS. Apoptosis was analyzed using flow cytometry. For cell cycle analysis, HASMCs were stained with PI for 30 min at room temperature after being fixed with 70% ethanol for an overnight at 4 °C. The G0-G1, S, and G2-M phase cell percentages were determined by flow cytometry (Becton, Dickinson and Company, New Jersey, USA).

### Signaling pathway screening

AD-related genes were screened using GeneCards (https://genealacart.genecards.org/). Genes co-expressed with LILRB4 were identified using the COXPRESdb database (https://coxpresdb.jp/). The Venn tool (https://bioinformatics.psb.ugent.be/webtools/Venn/) was used to identify shared genes. Kyoto Encyclopedia of Genes and Genomes (KEGG) enrichment analysis of the shared genes was performed using the DAVID database (https://david.ncifcrf.gov/).

### Statistical analysis

Three independent experiments were conducted. GraphPad Prism software (version 8.0) was used for the data analysis. The findings are reported in the form of the mean ± standard deviation (SD) of the measures. Statistical differences between two groups were evaluated using a t-test. For multiple group comparisons, ANOVA was used and Tukey’s multiple comparison test was performed. Statistical significance was set at *p* < 0.05.

### Ethical approval

The experiments conformed to the Guide for the Care and Use of Laboratory Animals. Animal study has been approved by the Animal Ethics Committee of First Affiliated Hospital of Gannan Medical University (LLSC2023-153). All methods are reported in accordance with ARRIVE guidelines.

## Results

### Knockdown of LILRB4 reduces AD formation

**Figure 1:**
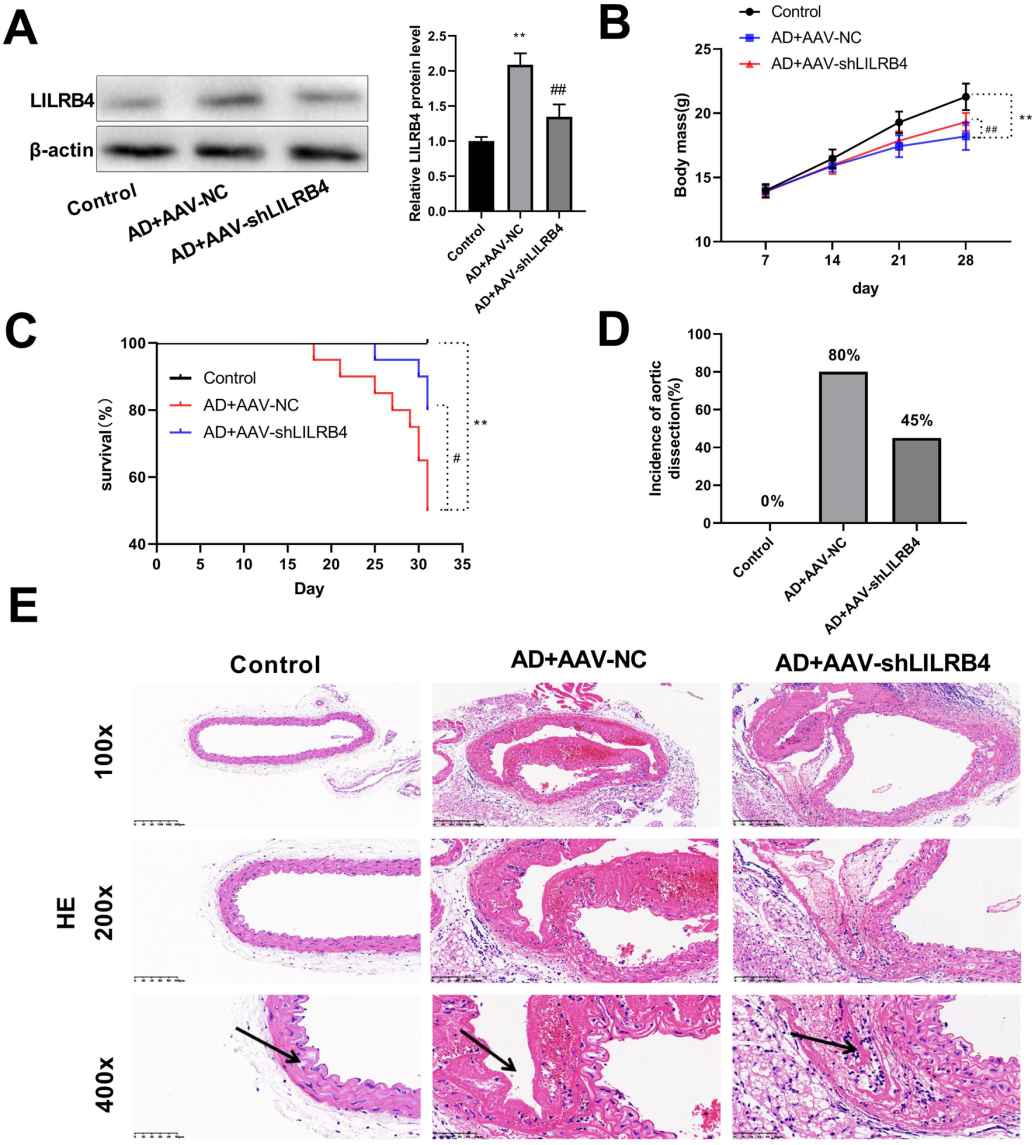
Knockdown of LILRB4 inhibits AD formation (n = 6). (**A**) AAV2-mediated knockdown of LILRB4 was evaluated using western blot analysis. (**B**) Changes in body weight of mice in control, AD + AAV-NC, and AD + AAV-shLILRB4 groups. (**C**) The survival rate of mice in the three groups. (**D**) Probability of AD formation in each group of mice. (**E**) HE staining of the aorta in the three groups. Scale bar: 400 μm. Data are represented as mean ± SD of six mice. Unpaired Student's t test: ^**^P < 0.01 versus Control group; ^#^*P* < 0.05, ^##^*P* < 0.01 versus AD + AAV-NC group.

An AD mouse model was established using BAPN in combination with Ang II induction. First, we examined the serum levels of ALT, AST, and ALP in liver tissues (Supplementary Fig. 1A); serum levels of BUN and Cr in kidney tissues (Supplementary Fig. 1B); serum levels of SOD, CAT, and MDA both in liver and kidney tissues (Supplementary Figs. 1C and D) to assess liver and kidney injury. The results showed that no significant differences were present in the levels of these serum markers between controls, AD + AAV-NC, and AD + AAV-shLILRB4 groups. Western blot results showed that the protein level of LILRB4 was significantly elevated in the AD mice model compared to that in the control group. In the AD + AAV-sh LILRB4 group, LILRB4 expression was significantly lower than that in the AD + AAV-NC group (Fig. 1A). A previous study has shown that BAPN treatment can reduce weight gain in FVB and C57BL/6 mice^22^. In this study, we found that the body weight of the BAPN and Ang II-induced C57BL/6 mice lowered and LILRB4 knockdown increased body weight of these AD mice (Fig. 1B). That the survival rate of mice in the AD + AAV-NC group was lower than that in the control group. LILRB4 knockdown prolonged the survival time and survival rate of AD mice (Fig. 1C). The incidence of AD was also assessed. Our findings revealed that the probability of AD occurrence in the AD + AAV-NC group was 80%, which decreased to 45% in the AD + AAV-shLILRB4 group (Fig. 1D). HE staining was used to examine pathological changes in the aorta. The results demonstrated that the aorta in the control group was intact, with a neatly arranged smooth muscle layer in the outer wall of the vessel (indicated by arrowheads) and no entrapment. Conversely, the outer wall of the vessel in the AD + AAV-NC group was significantly thickened with inflammatory cell infiltration and the outer wall of the vessel was ruptured in severe cases. In the AD + AAV-shLILRB4 group, false lumens were smaller than those in the AD + AAV-NC group and the overall vessel wall morphology was smoother than that in the model group (Fig. 1E).

### Knockdown of LILRB4 promotes contractile phenotype switch and ECM stability in AD mouse model

**Figure 2:**
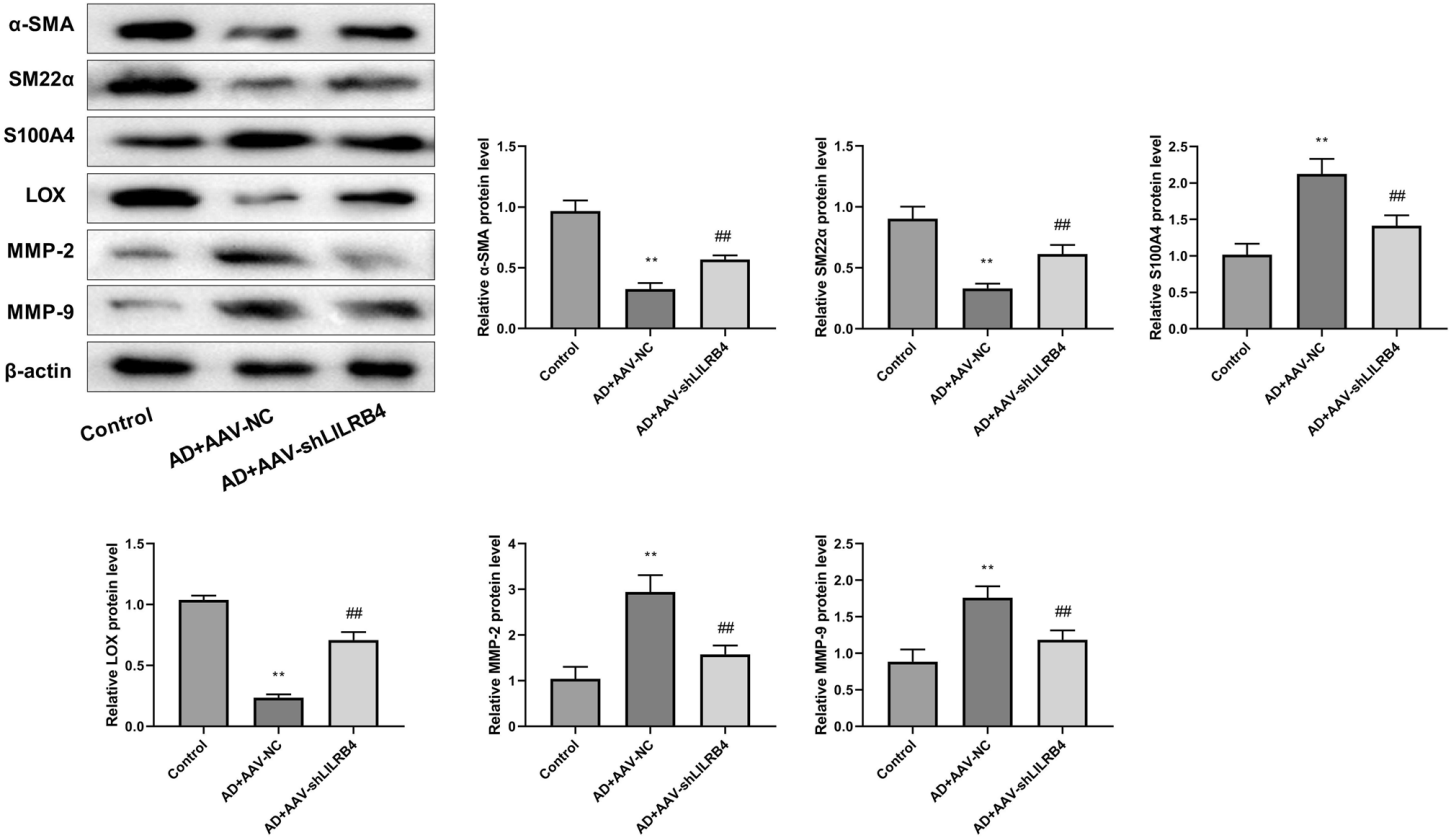
Expression of aortic phenotype transition-related proteins and ECM proteins in mice of different treatment groups (n = 6). Data are represented as mean ± standard deviation (SD) of six mice. Unpaired Student's t test: **P < 0.01 versus Control group; ^##^*P* < 0.01 versus AD + AAV-NC group.

Subsequently, we examined the levels of phenotype switch- and ECM-related proteins in mouse aortic tissues by western blot (Fig. 2). Our data indicated that the contractile proteins α-SMA and SM22α expressions were reduced, whereas the synthetic protein S100A4 was enhanced in the AD + AAV-NC group, suggesting that the cellular phenotype was converted from contractile to synthetic. This trend was reversed by LILRB4 knockdown. Furthermore, LILRB4 knockdown promoted LOX protein expression level and suppressed MMP2 and MMP9 expression, suggesting that LILRB4 knockdown promoted ECM stabilization.

### Knockdown of LILRB4 inhibits pyroptosis in AD mouse model

**Figure 3:**
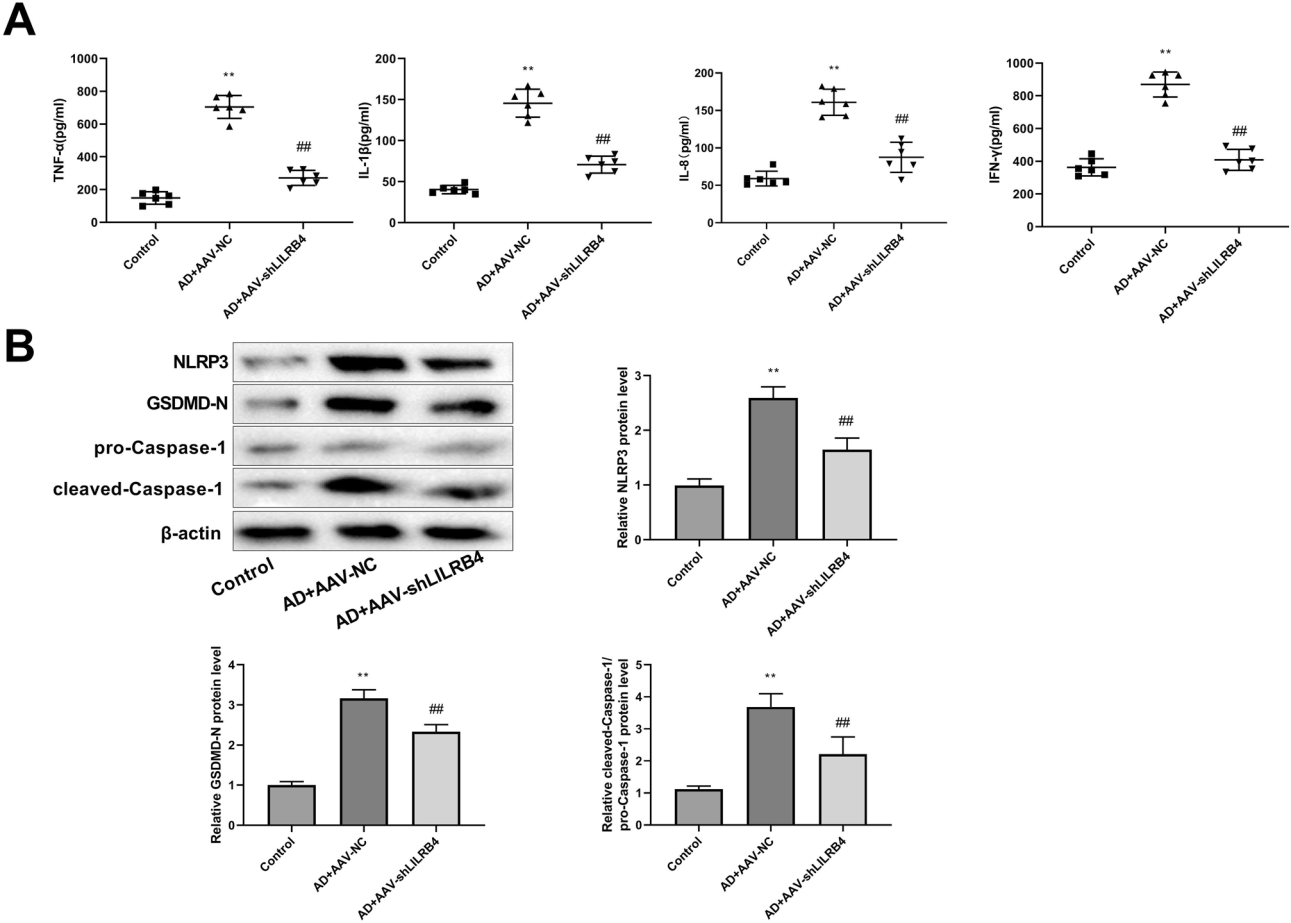
LILRB4 knockdown inhibited pyroptosis in AD mice (n = 6). (**A**) ELISA was performed to detect the levels of pro-inflammatory cytokines, including TNF-α, IL-1β, IL-8, and IFN-γ, in the serum of mice in each group. (**B**) Western blot was performed to measure the expression levels of pyroptosis-associated proteins, including NLRP3, GSDMD-N, and cleaved-caspase-1, in AD model mice. Data are represented as mean ± standard deviation (SD) of six mice. Unpaired Student's t test: ^**^P < 0.01 versus Control group, ^##^*P* < 0.01 versus AD + AAV-NC group.

The levels of pro-inflammatory cytokines in the serum of mice were detected using ELISA. The results suggested that the LILRB4 knockdown resulted in decreased expression levels of TNF-α, IL-1β, IL-8, and IFN-γ in AD model (Fig. 3A). Moreover, the expression levels of pyroptosis-related proteins, including NLRP3, GSDMD-N, and cleaved-caspase-1 were significantly elevated in the AD model mice relative to those in the control group and knockdown of LILRB4 substantially reduced these protein levels (Fig. 3B).

### Knockdown of LILRB4 promotes contractile phenotype switch and ECM stability in AD cell models

**Figure 4:**
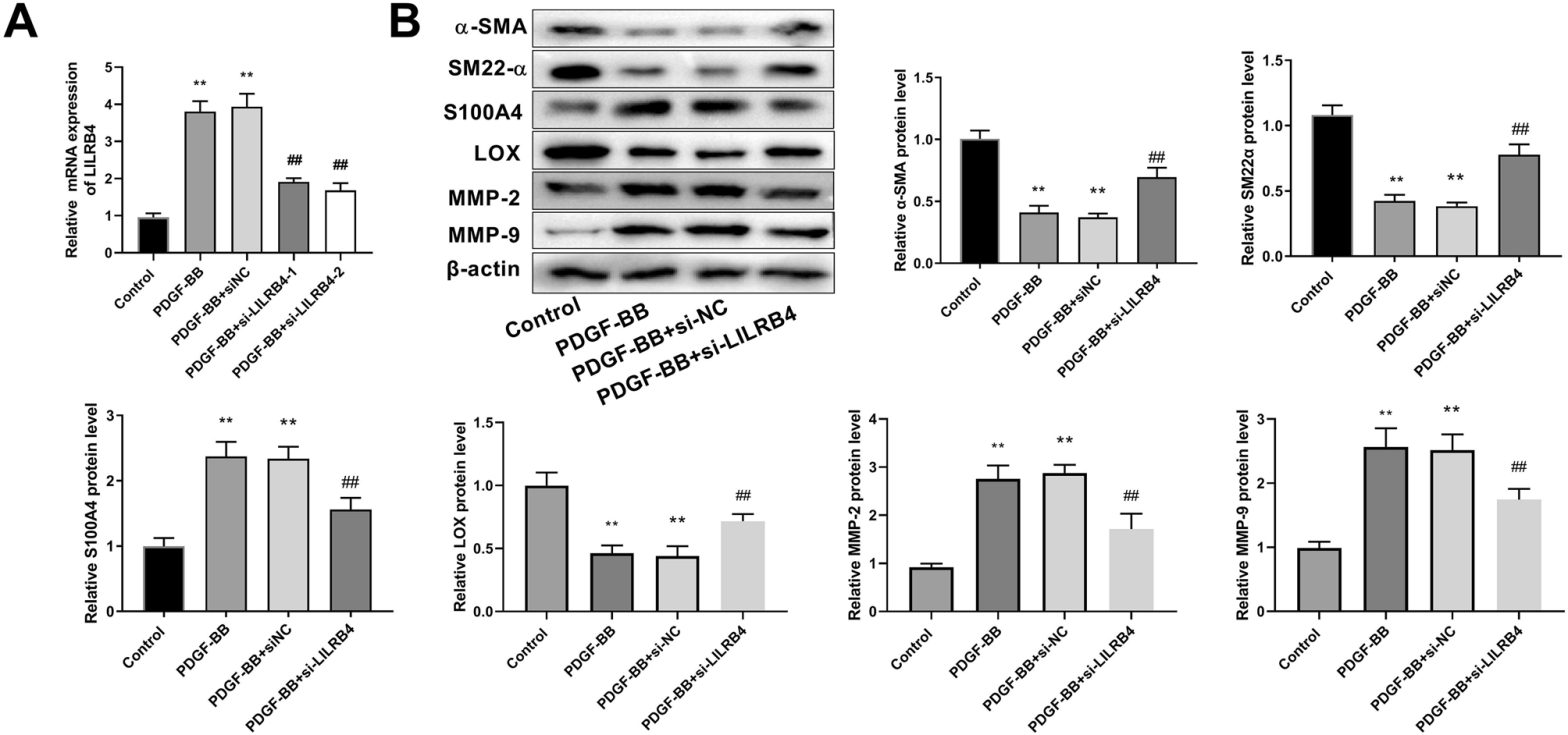
LILRB4 knockdown promotes contractile phenotype switching and ECM stabilization in AD cell models. (**A**) The mRNA level of LILRB4 in the control, PDGF-BB, PDGF-BB + siNC, PDGF-BB + si-LILRB4-1, and PDGF-BB + si-LILRB4-2 groups was detected by RT-qPCR. The data are expressed as mean ± standard deviation (SD) from three biological replicates. One way ANOVA followed by Tukey’s post-hoc test: ^**^P < 0.01 vs. Control group, ^##^*P* < 0.01 versus PDGF-BB + si-NC group. (**B**) Western blot analysis was conducted to assess the levels of contractile proteins, including α-SMA and SM22α, synthetic proteins S100A4, as well as ECM-related proteins LOX, MMP2, and MMP9 in the control, PDGF-BB, PDGF-BB + si-NC, and PDGF-BB + si-LILRB4 groups. Data are represented as mean ± standard deviation (SD) of three independent experiments. One way ANOVA followed by Tukey’s post-hoc test: ^**^P < 0.01 versus Control group; Unpaired Student's t test: ^##^*P* < 0.01 versus PDGF-BB + si-NC group.

HASMCs were treated with PDGF-BB to generate an in vitro AD model. RT-qPCR results demonstrated that the mRNA level of LILRB4 was significantly higher in the in vitro model of AD. After transfection with si-LILRB4-1 and si-LILRB4-2, the expression level of LILRB4 was markedly decreased (Fig. 4A). The si-LILRB4-2 was used for further experiments. Western blot revealed that PDGF-BB treatment lowered α-SMA and SM22α protein levels, whereas increased the synthetic protein S100A4 expression, which was notably overturned by the LILRB4 knockdown (Fig. 4B). Thus, LILRB4 downregulation suppressed the effect of PDGF-BB on the phenotypic transformation of HASMCs. In addition, LOX expression levels decreased, and MMP2 and MMP9 levels were upregulated upon PDGF-BB induction. LILRB4 downregulation enhanced LOX protein expression and decreased MMP2 and MMP9 protein expression (Fig. 4B).

### Knockdown of LILRB4 inhibits the JAK2/STAT3 signaling pathway

**Figure 5:**
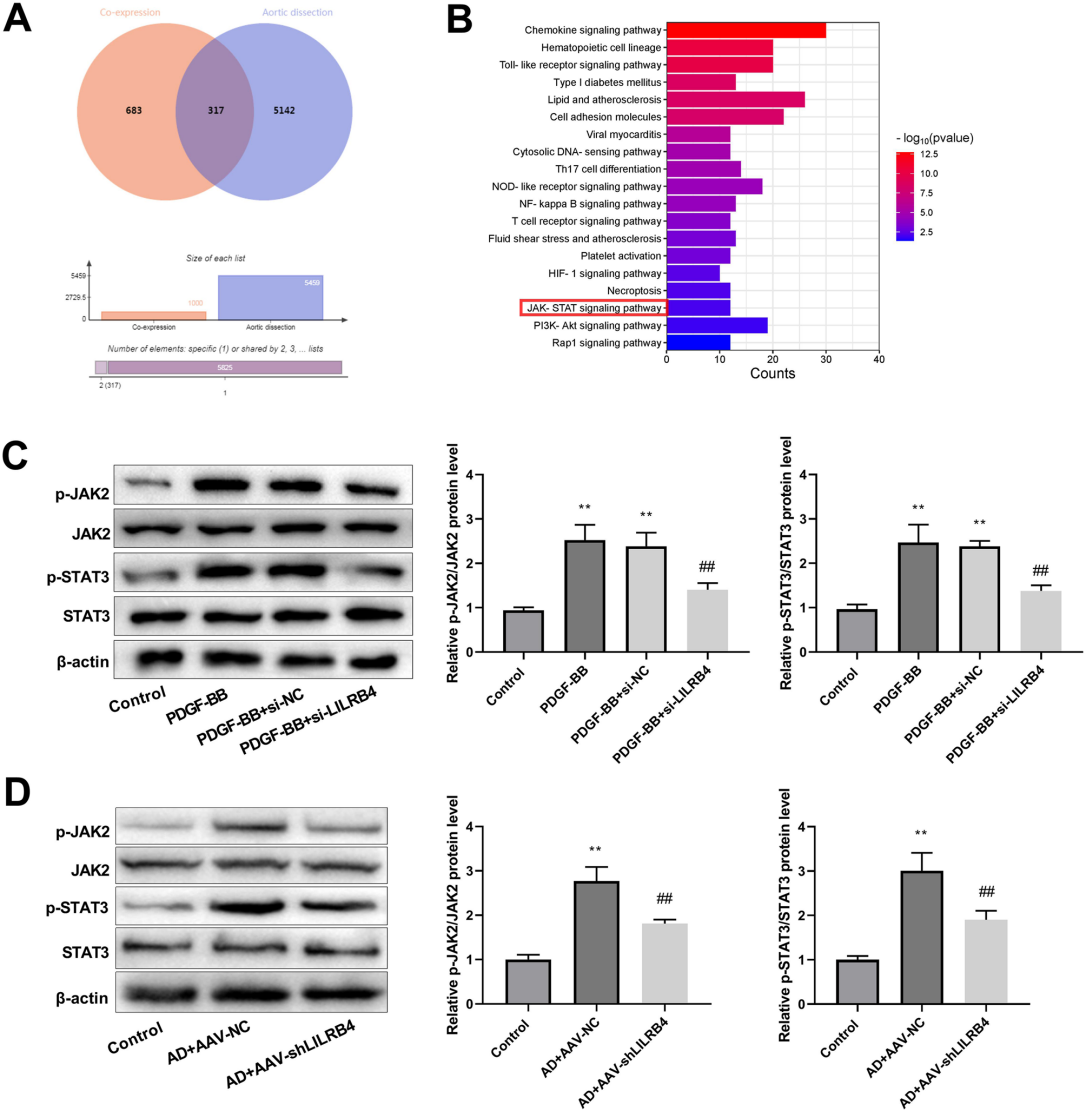
Downstream pathway analysis of LILRB4. (**A**) Venn diagram depicting the intersection of the top 1000 co-expressed genes of LILRB4 with AD-related genes. (**B**) KEGG enrichment analysis was conducted on the common genes to identify enriched biological pathways. (**C**) Western blot was used to detect the expression of proteins associated with the JAK2/STAT3 signaling pathway, including p-JAK, JAK, p-STAT, and STAT in the control, PDGF-BB, PDGF-BB + si-NC, and PDGF-BB + si-LILRB4 groups. The data are expressed as mean ± standard deviation (SD) from three independent experiments. One way ANOVA followed by Tukey’s post-hoc test: ^**^P < 0.01 versus Control group; Unpaired Student's t test: ^##^*P* < 0.01 *vs*. PDGF-BB + si-NC group. (**D**) The phosphorylation levels of JAK2 and STAT3 proteins were measured by western blot in the AD mouse model (n = 6). The data are expressed as mean ± standard deviation (SD) of six mice. Unpaired Student's t test: ^**^P < 0.01 versus Control group; ^##^*P* < 0.01 versus AD + AAV-NC group.

The potential signaling pathways involving LILRB4 in AD were also explored. The Coxpresdb database was used to screen the top 1000 co-expressed genes with LILRB4, whereas AD-related genes were extracted from the GeneCards database. The Venn diagram illustrates that 317 overlapping genes (Fig. 5A) were significantly enriched in the JAK/STAT pathway (Fig. 5B). Western blot demonstrated that JAK2 and STAT3 phosphorylation (p-JAK2 and p-STAT3) levels were elevated in the PDGF-BB and PDGF-BB + siNC groups. These protein levels in the PDGF-BB + si-LILRB4 group were lower than those in the PDGF-BB + si-NC group (Fig. 5C). Similarly, in the AD mouse model, the p-JAK2/JAK2 and p-STAT3/STAT3 values were significantly reduced after LILRB4 knockdown (Fig. 5D), indicating that LILRB4 silencing inhibited JAK2/STAT3 pathway activation.

### JAK2/STAT3 signaling pathway inhibitor suppresses AD cell development

**Figure 6:**
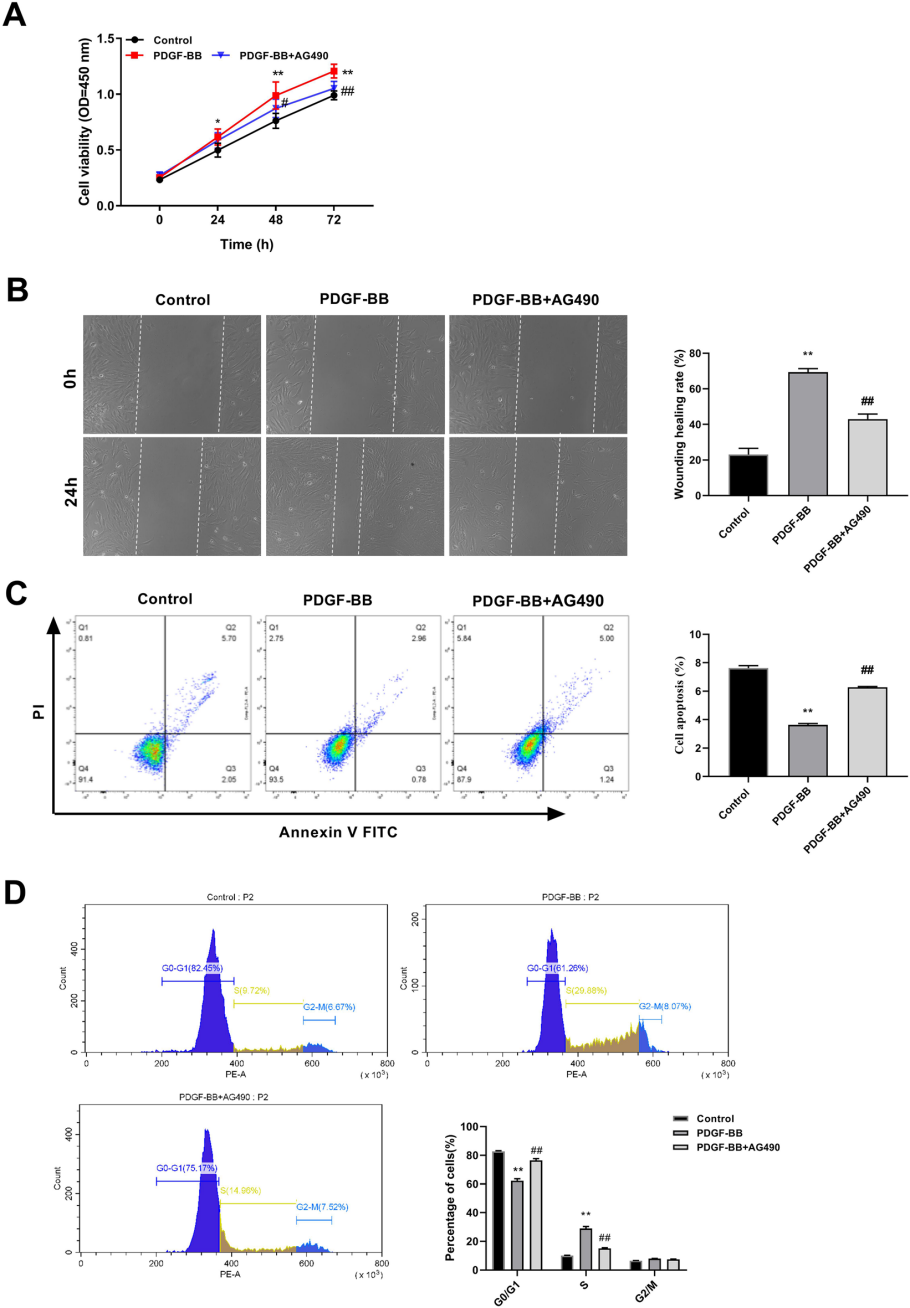
Impact of JAK/STAT signaling pathway on PDGF-BB-induced HASMC function. (**A**) The CCK-8 assay was used to investigate the effect of the JAK2/STAT3 signaling pathway inhibitor AG490 on PDGF-BB-induced HASMC viability. (**B**) The impact of on migration was assessed using the wound healing assay. (**C–D**) Flow cytometry was employed to evaluate apoptosis and cell cycle distribution after AG490 treatment. The data are expressed as mean ± standard deviation (SD) of three biological replicates. Unpaired Student's t test: ^**^P < 0.01 versus Control group; ^##^*P* < 0.01 versus PDGF-BB group.

To further investigate the function of JAK2/STAT3 pathway in AD, PDGF-BB-induced HASMCs were treated with AG490 (10 µM), an inhibitor of the JAK2/STAT3 signaling pathway. The CCK-8 results revealed a significant increase in cell viability upon PDGF-BB treatment compared to that in the control group and this trend was notably reversed by the incorporation of the AG490 inhibitor (Fig. 6A). The results of the wound-healing assay demonstrated that PDGF-BB treatment promoted cell migration. Concurrently, AG490 addition significantly inhibited PDGF-BB-induced HASMCs migration (Fig. 6B). According to the results of flow cytometry analysis, AG490 promoted apoptosis in PDGF-BB-induced AD cells (Fig. 6C). Furthermore, the G0/G1 phase ratio of HASMCs was reduced and the S phase ratio was augmented upon PDGF-BB treatment, which was significantly reversed by AG490 treatment (Fig. 6D). Altogether, AG490, an inhibitor of the JAK/STAT signaling pathway, reduced AD cell viability and migration, promoted apoptosis, and caused a notable increase in the G0/G1 phase ratio while decreasing the S phase ratio.

### JAK2/STAT3 signaling pathway inhibitor facilitates contractile phenotype switch and ECM stability in AD cell model

**Figure 7:**
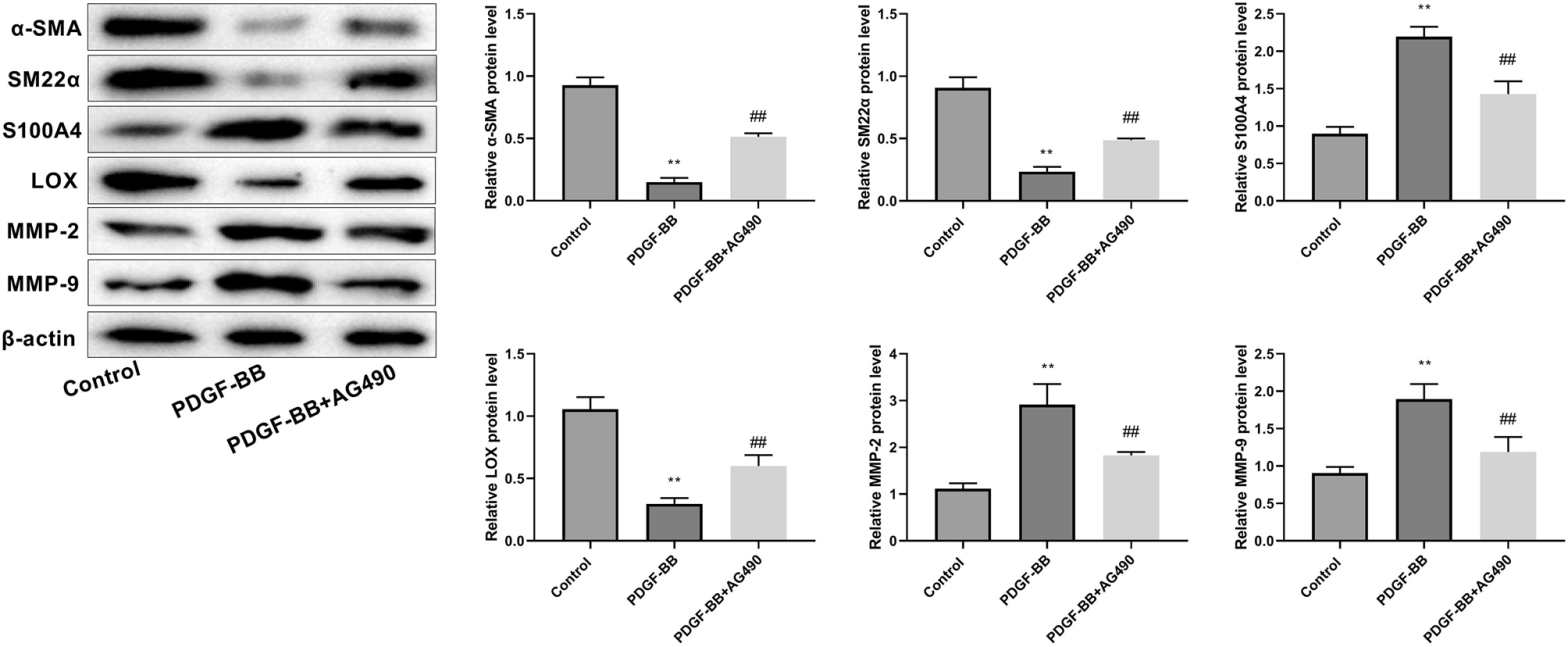
The JAK/STAT signaling pathway inhibitor AG490 promotes AD contraction phenotypic shift and ECM stability in AD cell model. The data are expressed as mean ± standard deviation (SD) of three independent experiments. Unpaired Student's t test: ^**^P < 0.01 versus Control group; ^##^*P* < 0.01 versus PDGF-BB group.

The inhibitor AG490 effectively reversed the downregulation of contractile proteins (α-SMA and SM22α) and the upregulation of synthetic protein (S100A4) expression that were induced by PDGF-BB stimulation. These findings suggested that AG490 reversed the transformation of the cell phenotype from contractile to synthetic. Additionally, AG490 increased LOX protein expression and decreased MMP2 and MMP9 expression in AD cells (Fig. 7), suggesting that the JAK/STAT inhibitor promoted ECM stabilization.

## Discussion

AD is a critical medical emergency and its exact pathogenesis remains unclear. Mutations in LOX are considered pivotal in AD development^23^. BAPN, a LOX inhibitor, elicits AD in mice, which exhibits a high degree of similarity to human pathogenesis^24,25^. Currently, the two most common AD animal models involve Ang II administration through subcutaneous injection or pretreatment with BAPN^26^. In this study, we constructed an AD mouse model using a combination of BAPN and Ang II and an AD cell model by inducing HASMCs with PDGF-BB. Our findings revealed that LILRB4 knockdown promoted the contractile phenotype and ECM stabilization, and inhibited pyroptosis and the JAK/STAT pathway.

LILRB4, also known as ILT3, is an essential immune checkpoint molecule that maintains immune homeostasis and modulates immune responses. LILRB4 is associated with several diseases, including cancer, autoimmune diseases, chronic inflammation, infectious diseases, and transplantation ^27–31^. LILRB4 is highly expressed in acute myeloid leukemia and mediates T-cell suppression and infiltration^32^. Moreover, LILRB4-targeting antibody–drug conjugates show great clinical potential for the treatment of acute myeloid leukemia^33^. LILRB4 dysregulation boosts pro-inflammatory cytokine production, diminishes IL-10 levels in B cells, and regulates T- and B cell-mediated autoimmune diseases such as systemic lupus erythematosus^34^. In addition, abnormal LILRB4 correlates significantly with inflammatory diseases, such as multiple sclerosis and inflammatory bowel disease^35,36^. However, the function and molecular mechanisms of LILRB4 in AD remain unclear. The present study demonstrated that LILRB4 knockdown inhibited AD progression.

Vascular smooth muscle cells (vSMCs) are crucial for the aortic wall structure, functional integrity, and ECM formation. The plasticity of vSMCs allows them to switch between the contractile and synthetic phenotypes in response to environmental stimuli and mechanical stress^37^. Most vSMCs mediators maintain normal arterial function by maintaining a contractile phenotype, regulating vascular tone, and sustaining hemodynamic balance^38^. In AD, the balance between contractile and synthetic vSMCs shifts in favor of synthetic vSMC. This shift is accompanied by the excessive production of protein hydrolases that subsequently degrade the ECM^39^. Moreover, synthetic vSMCs exhibit enhanced proliferation and migration. The expressions of contractile proteins, such as SM22α and α-SMA were reduced^40,41^. In this study, we found that the expression levels of α-SMA and SM22α, were significantly down-regulated, whereas the expression of the synthetic protein S100A4 was up-regulated in AD, which was reversed by knockdown of LILRB4. Previous studies have demonstrated that multiple genes affect AD development by modulating the phenotypic switching of vSMCs. METTL3 inhibits the contractile phenotype of HASMCs and promotes AD by enhancing the m6A modification of NOTCH1 and repressing NOTCH1^42^. HIF-1a induces phenotype switch of HASMCs by regulating PI3K/AKT/AEG-1 signaling pathway^43^. In this study, we found that LILRB4 knockdown promoted the AD contractile phenotype and impeded the development of AD.

Local expression and elevated activity of metalloproteinases (MMPs) promote elastic fiber fracture, aortic dilatation, ECM degeneration, and vascular framework disruption^44,45^. MMP-2 and MMP-9 are the two most prevalent MMPs associated with AD development^46^. MMP-2 and MMP-9 expressions are elevated in AD^47^. Overexpression of MMP2 and MMP9 degrades ECM components, including collagen and elastin to weaken the aortic wall and exacerbating aorta narrowing^48,49^. ROS production and elevated levels of HIF-1α triggered by intermittent hypoxia and reoxygenation exposure contribute to the worsening of AD by promoting the production of MMP2, MMP9, and vascular endothelial growth factor^50^. Upregulation of MMP2 and MMP9 triggered by BRG1 promotes HASMCs apoptosis and synthetic phenotypes, which in turn foster the development of thoracic AD^51^. In the current study, MMP2 and MMP9 were significantly upregulated in the AD model and their expression significantly decreased when LILRB4 was knocked down.

Pyroptosis, a form of programmed cell death mediated by the caspase family, triggers the release of various proinflammatory cytokines and contributes to inflammation and immune responses^52^. The expression of NLRP3, cleaved-caspase-1 and GSDMD-N was observed to be upregulated in AD^8,53^. Our study revealed that NLRP3, caspase-1, and GSDMD-N levels increased in the AD model, which was consistent with previous research. The dysregulation of NLRP3 and caspase-1 induces SMCs contractile dysfunction, leading to myosin heavy chain degradation^54^. Additionally, deletion of NLRP3 or caspase-1 reduces aortic dilatation and inhibits AD development^55^. Upon caspase-1 activation, precursor forms of IL-1β and IL-18 are processed to their mature active state^56^. Pro-inflammatory cytokines, such as TNF-α, IL-1β, IL-8, and IFN-γ, play important roles in the formation and development of AD^57^. In this investigation, LILRB4 knockdown suppressed the protein expression of NLRP3, GSDMD-N, and cleaved-caspase-1 and reduced the expression levels of TNF-α, IL-1β, IL-8 and IFN-γ. Moreover, most studies have shown that inhibitors targeting key proteins involved in pyroptosis are promising for AD treatment. NLRP3 inhibitors, such as glyburide and MCC950, inhibit AD formation^53,58^. This suggests that LILRB4 has the potential to act as a therapeutic target for AD by regulating pyroptosis.

The JAK/STAT signaling pathway, composed of JAK and the signal transducer and activator of STAT, participates in numerous biological processes. JAK2/STAT3 is a classical pathway responsible for the transcriptional activation and signal transduction of STAT^59^. Upon activation, the JAK/receptor complex facilitates the phosphorylation of STAT proteins, which then translocate to the nucleus and regulate the expression of downstream target genes^60,61^. In AD-induced acute lung injury, IL-22 inhibits Ang II-mediated pulmonary microvascular endothelial cell apoptosis and downregulates STAT3 expression and intranuclear delivery. Notably, this effect was significantly reversed by the addition of the JAK2 inhibitor AG490^62^. In the current study, LILRB4 knockdown suppressed the phosphorylation of JAK2 and STAT3. The JAK/STAT pathway inhibitor AG490 significantly inhibited the viability, migration, and S-phase cell cycle of PDGF-BB-induced HASMCs, and promoted apoptosis, contractile switching, and ECM stability.

This study had some limitations. First, immunostaining for ECM proteins (LOX, MMP9, and MMP2) and inflammatory genes was not conducted because of scientific constraints, which limited the comprehensive characterization of molecular changes. Second, the findings of this investigation were primarily based on BAPN- and Ang II-induced AD mouse models as well as a PDGF-BB-stimulated AD cell model. Further validation through clinical studies is necessary to confirm the relevance of these findings to human AD pathology.

## Conclusions

In this study, we found that LILRB4 silencing suppresses AD development by promoting contractile phenotype switch of HASMCs, enhancing ECM stability, and inhibiting pyroptosis, potentially by regulating the JAK2/STAT3 signaling pathway. Overall, our study provides novel potential therapeutic targets for AD treatment.

### Supplementary Information

Supplementary Information 1.

Supplementary Information 2.

Supplementary Information 3.

## Data Availability

The datasets used and/or analysed during the current study are available from the corresponding author on reasonable request.
