# Supplementary material for: LILRB4 knockdown inhibits aortic dissection development by regulating pyroptosis and the JAK2/STAT3 signaling pathway
Source: Sci Rep. 2024 Jul 6;14:15564. doi: 10.1038/s41598-024-66482-3 (PMC11227527; doi:10.1038/s41598-024-66482-3)

Supplementary figure 1 Detection of liver injury and kidney injury in AD mice (n = 6). A. Serum levels of ALT, AST, and ALP in liver tissues were detected in the three groups. B. Serum levels of BUN and Cr in kidney tissues were measured. C-D. SOD, CAT, and MDA in mouse serum in the liver tissues (C) and kidney tissues (D) were assessed using an automated biochemical analyzer (The data are expressed as mean ± standard deviation (SD) of six mice.


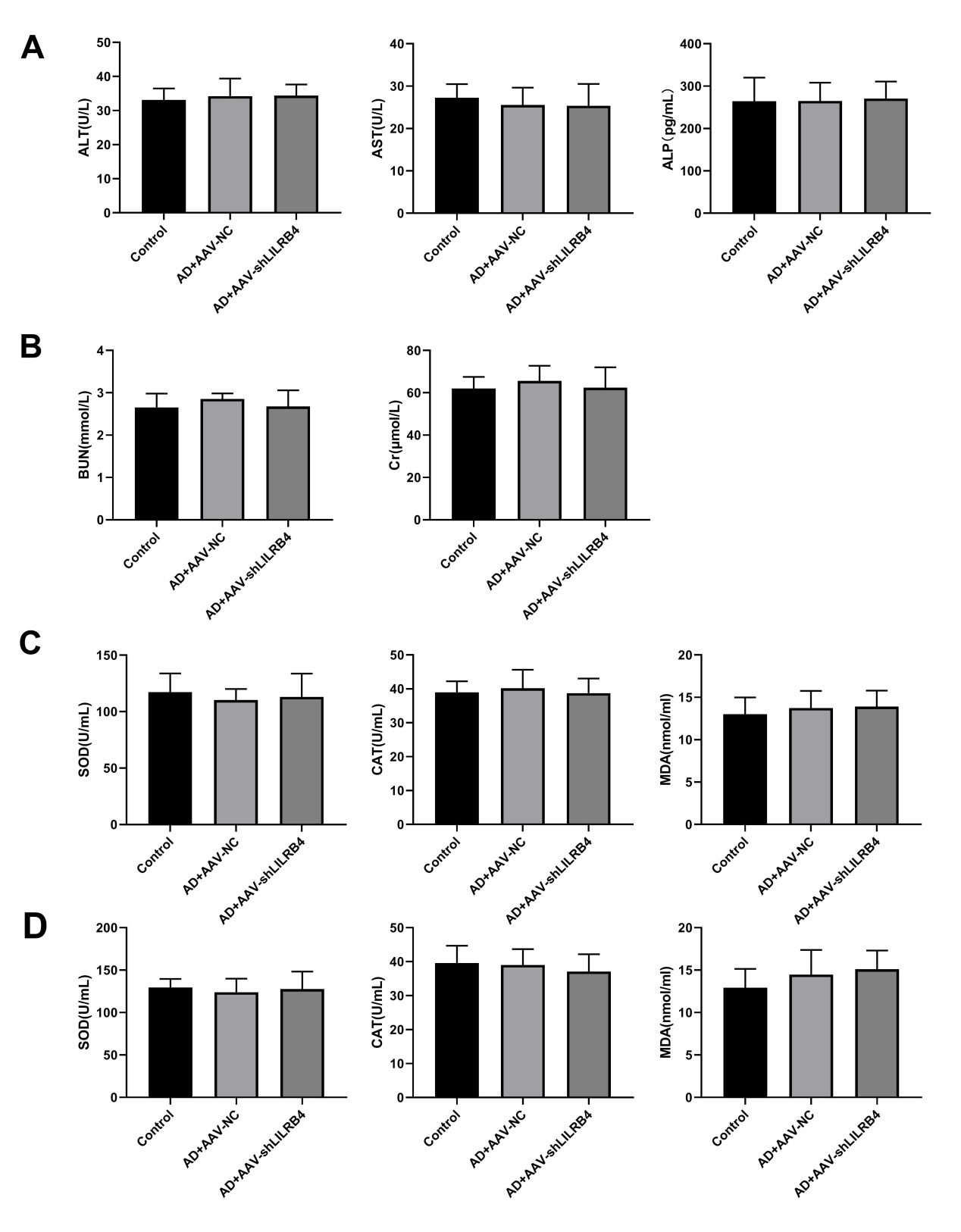

Supplement: Supplementary file 1 — Supplementary Information 1. [file 41598_2024_66482_MOESM1_ESM.docx]
