# Supplementary material for: LILRB4 knockdown inhibits aortic dissection development by regulating pyroptosis and the JAK2/STAT3 signaling pathway
Source: Sci Rep. 2024 Jul 6;14:15564. doi: 10.1038/s41598-024-66482-3 (PMC11227527; doi:10.1038/s41598-024-66482-3)
Supplement: Supplementary file 2 — Supplementary Information 2. [file 41598_2024_66482_MOESM2_ESM.docx]

Supplementary Table 1 Primer sequence information used in this study

| Name | Sense | Antisense |
| --- | --- | --- |
| siNC | UUCUCCGAACGUGUCACGUTT | ACGUGACACGUUCGGAGAATT |
| si-LILRB4-1 | GCAAUUCUAUGCUGACAUAAC | UAUGUCAGCAUAGAAUUGCUG |
| si-LILRB4-2 | CCUGAUUCUGCAAAGAUAAAU | UAGUGCUUAUAGCUCUUCCUG |
| GAPDH | AACTTTGGCATTGTGGAAGG | ACACATTGGGGGTAGGAACA |
| LILRB4 | ACCCACTGAAGATGGACTGG | TTGTGGGTTCCAACTGTTCA |
