# Supplementary material for: LILRB4 knockdown inhibits aortic dissection development by regulating pyroptosis and the JAK2/STAT3 signaling pathway
Source: Sci Rep. 2024 Jul 6;14:15564. doi: 10.1038/s41598-024-66482-3 (PMC11227527; doi:10.1038/s41598-024-66482-3)

Figure1A LILRB4

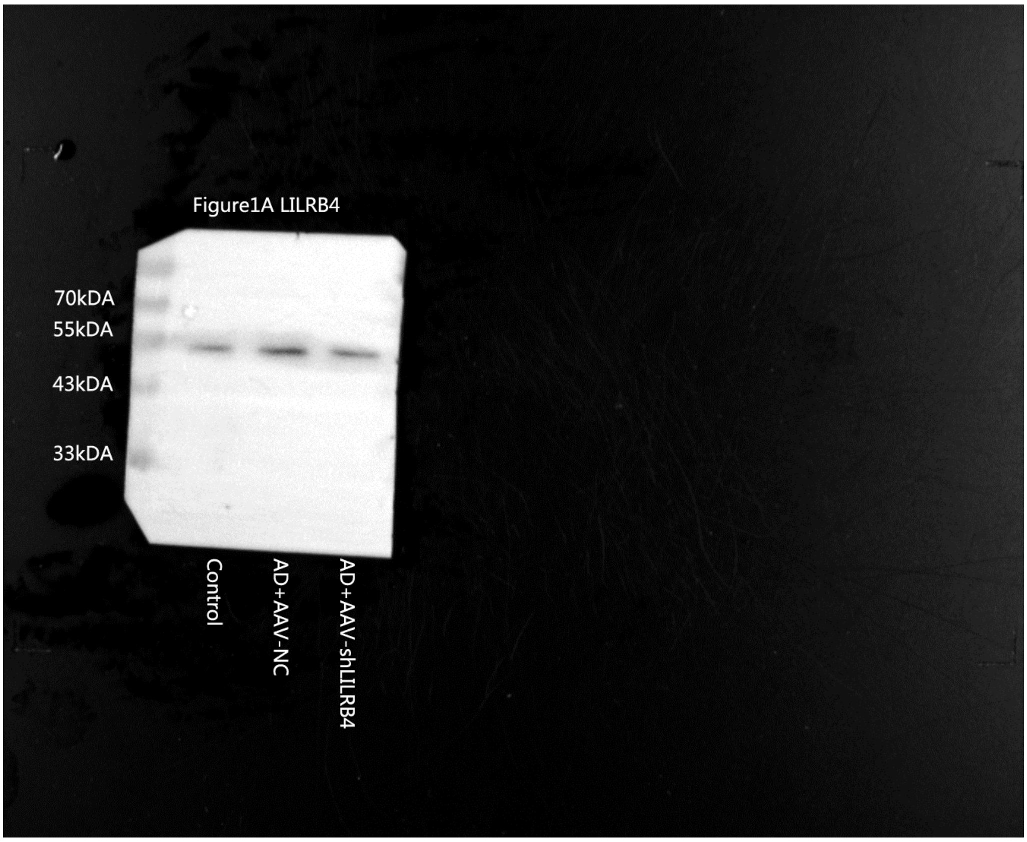

Figure1A β-actin

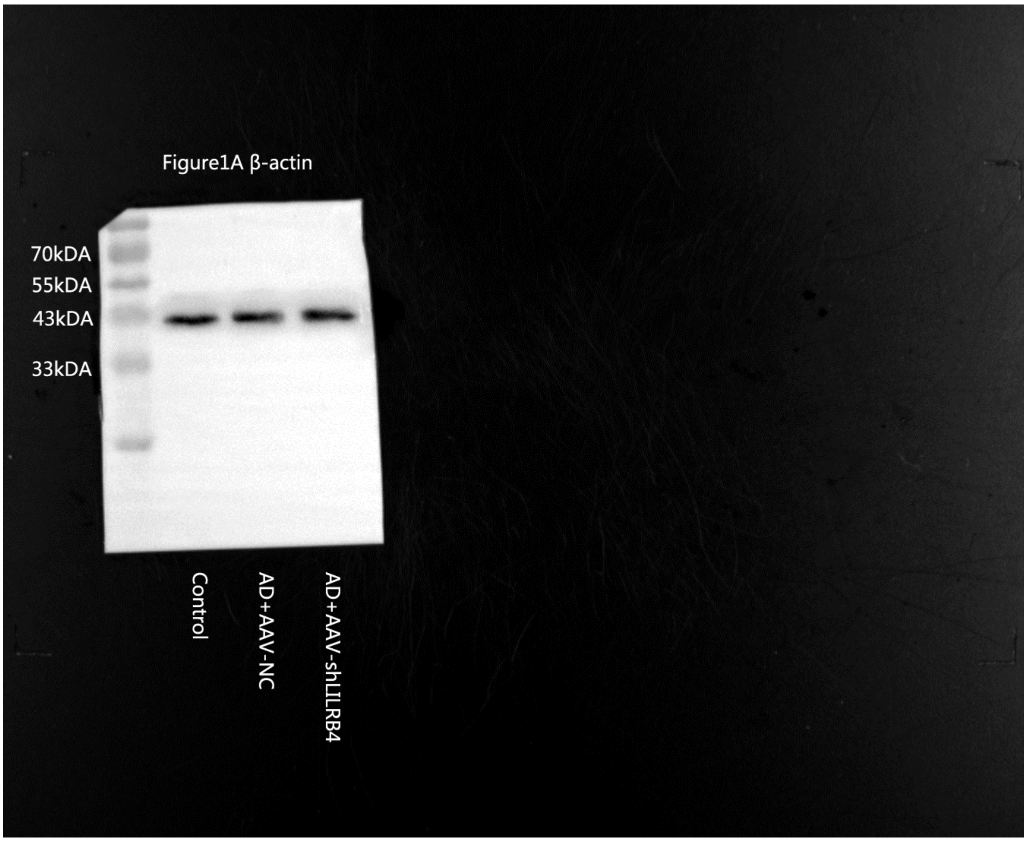

Figure2 LOX

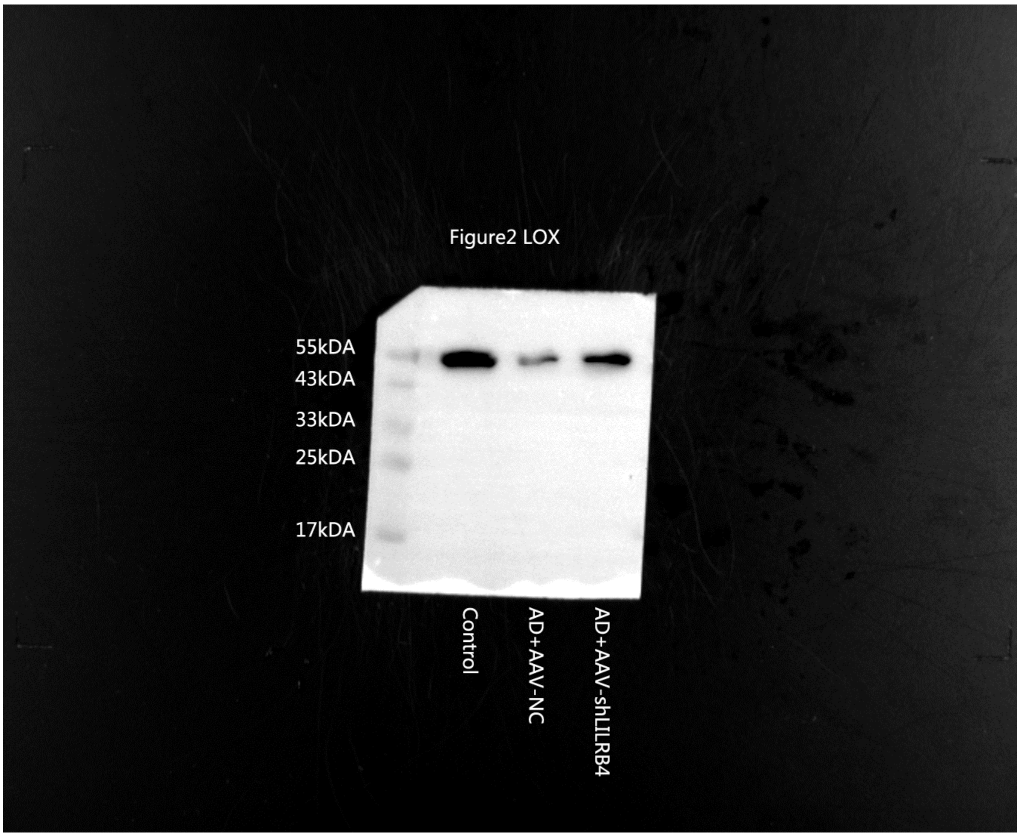
Figure2 LOX
55kDA
43kDA
33kDA
25kDA
17kDA
Control
AD+AAV-NC
AD+AAV-shLILRB4

Figure2 MMP-2

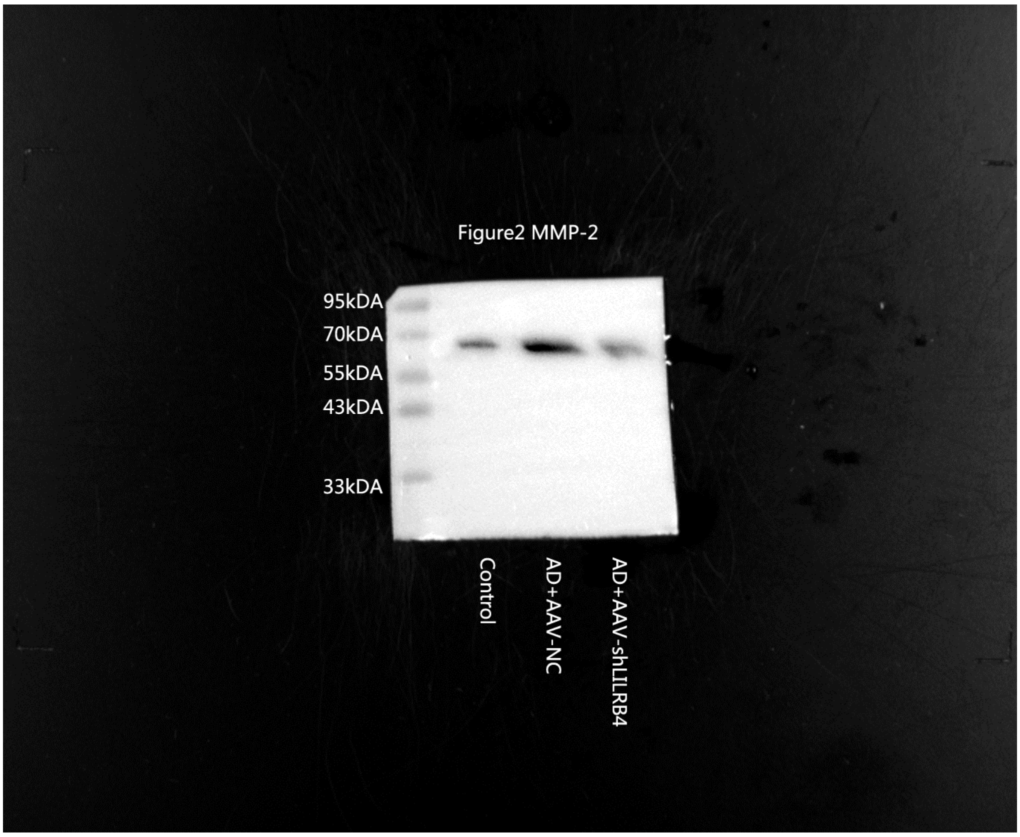

Figure2 MMP-9(Contains multiple exposures)

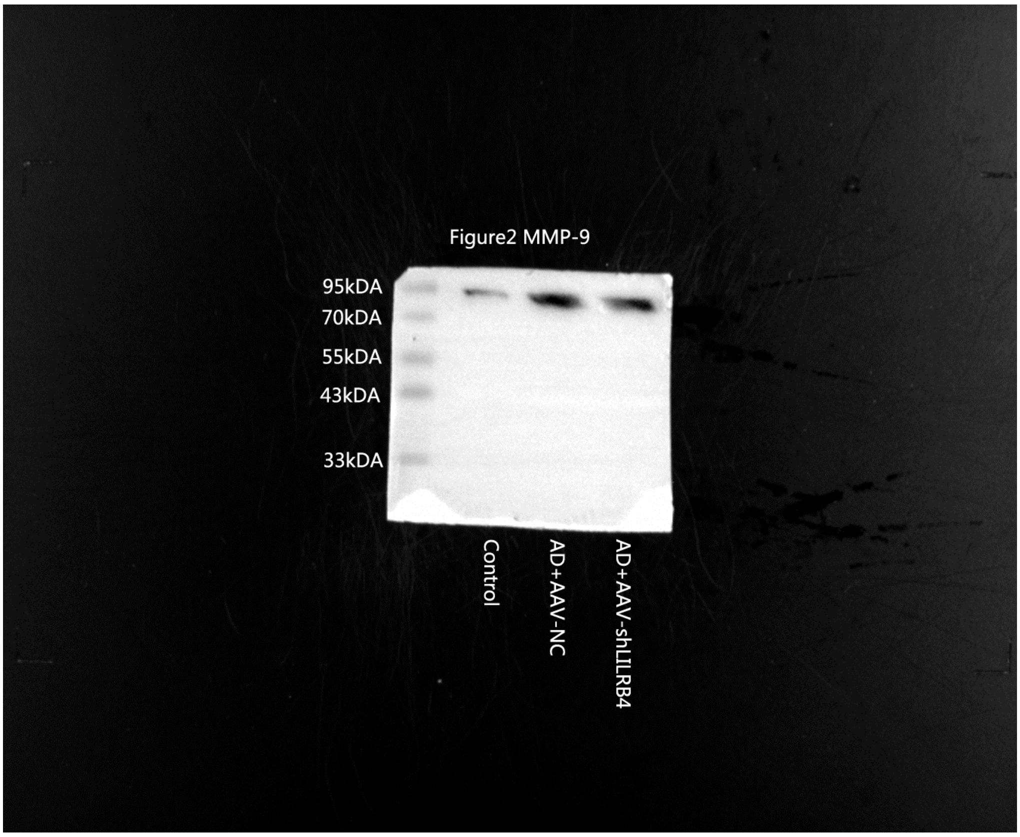

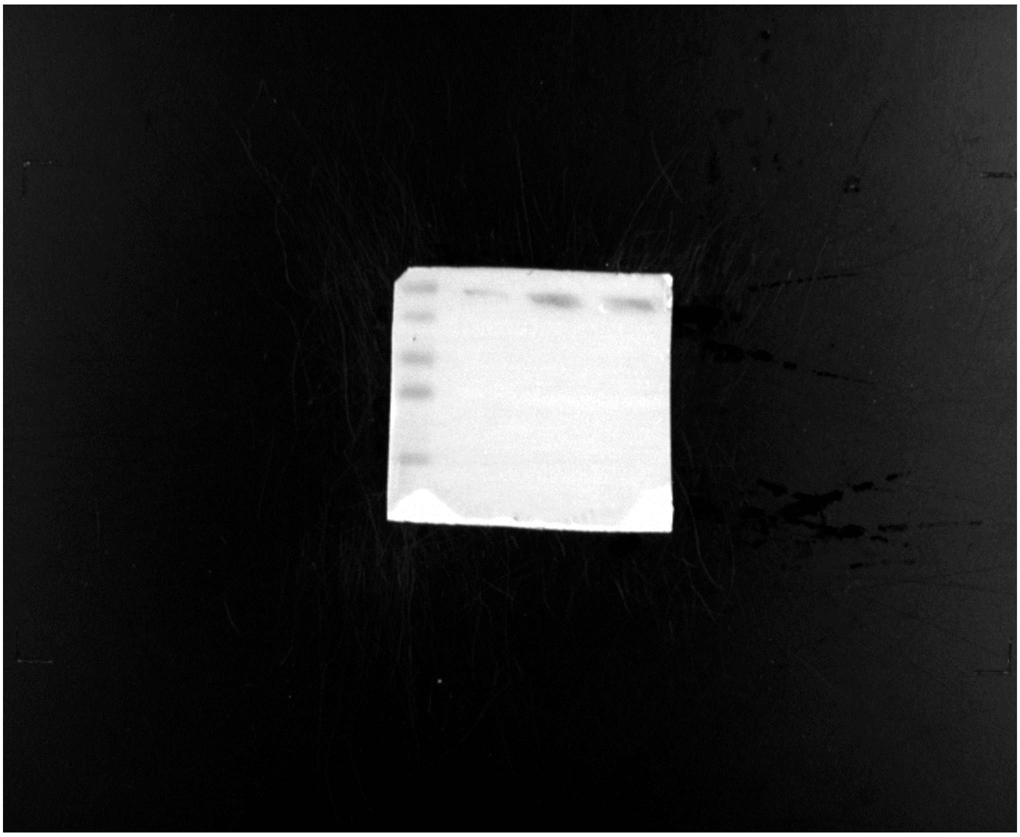

Figure2 SM22α (Contains multiple exposures)

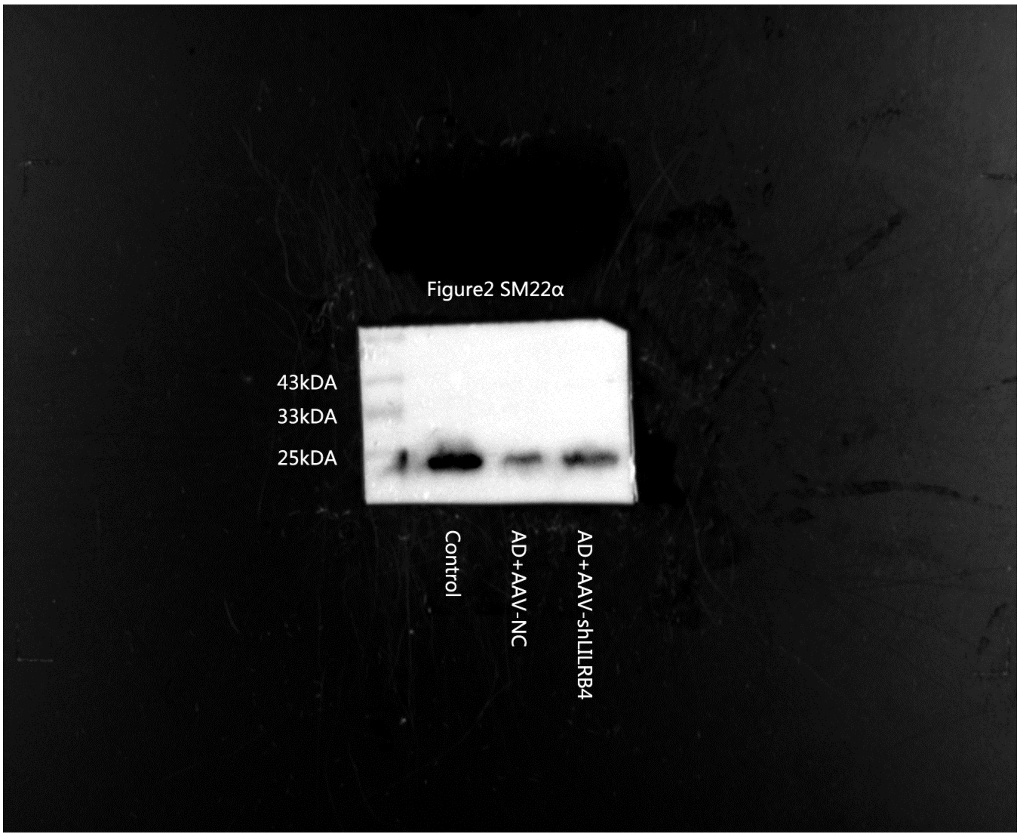

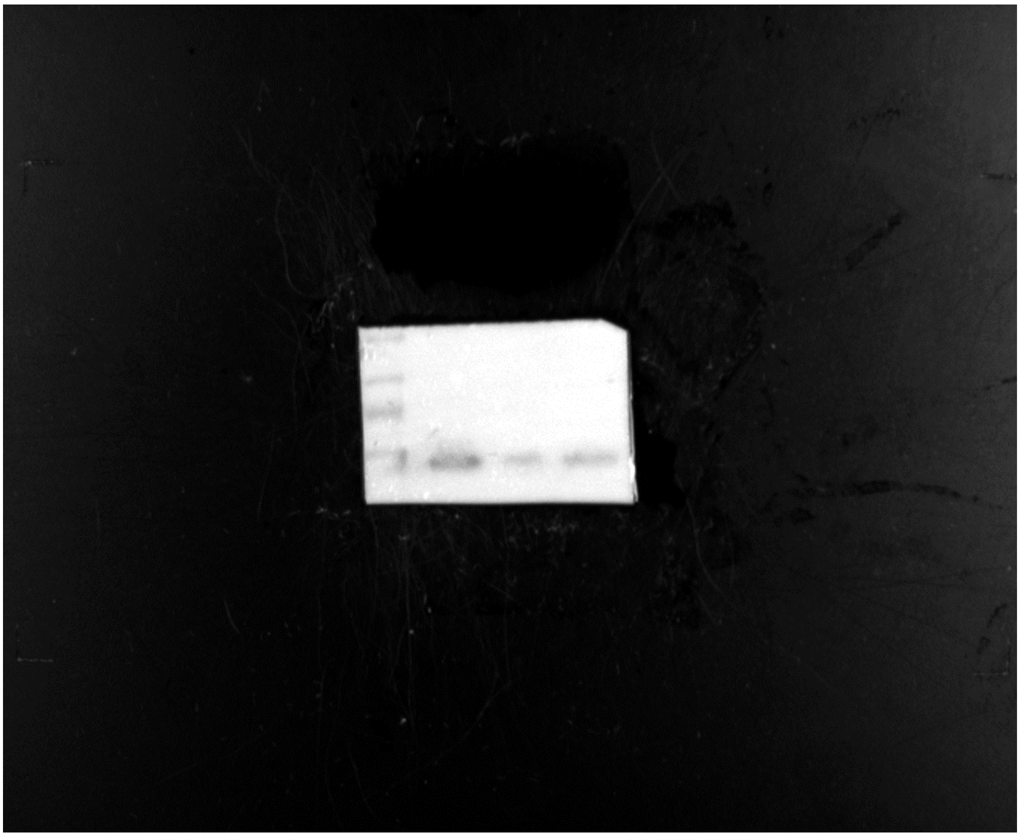

Figure2 α-SMA (Contains multiple exposures)

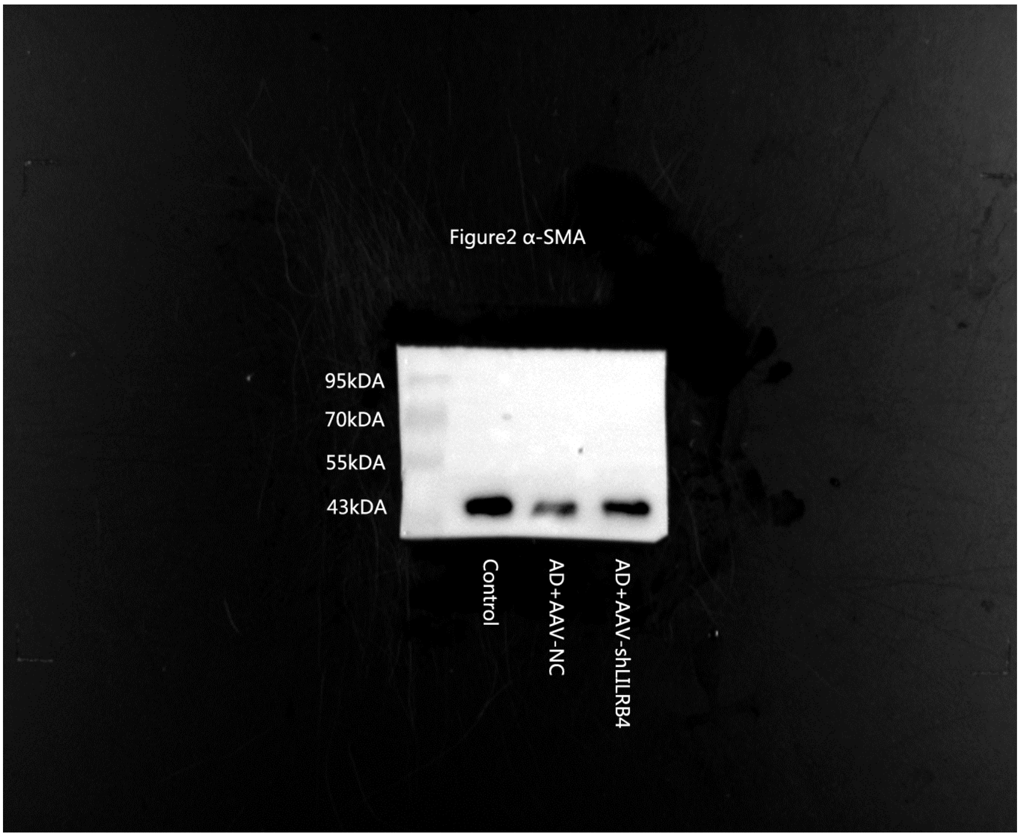

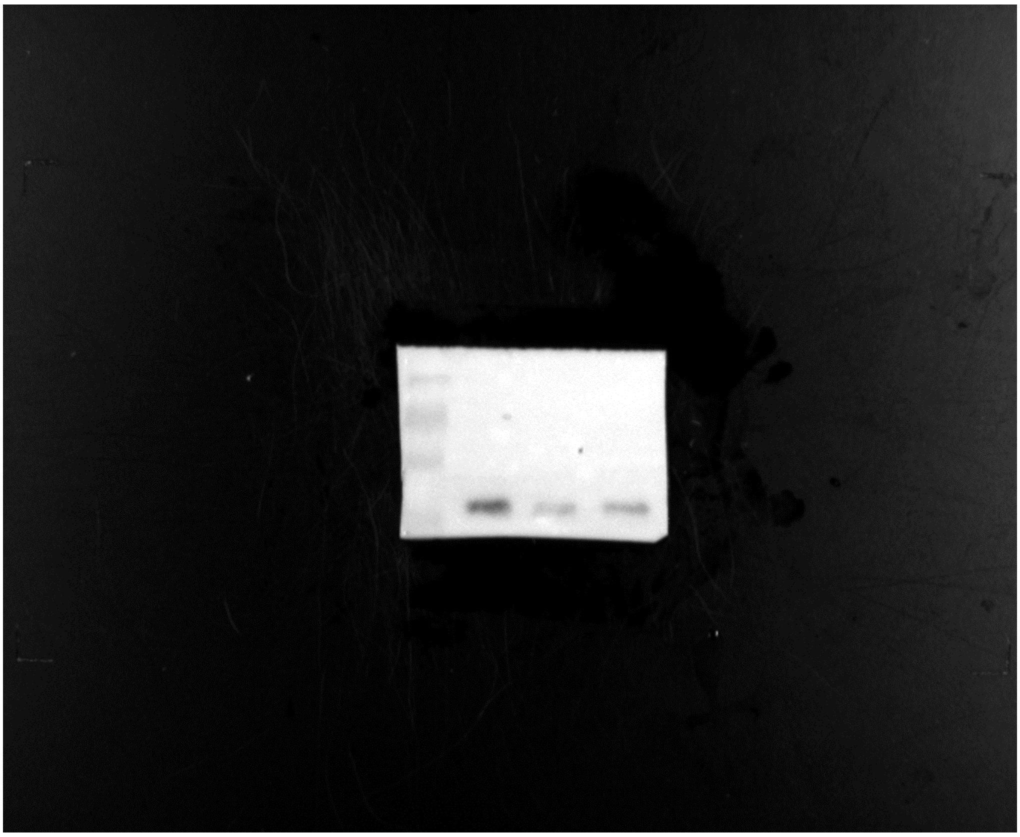

Figure2 β-actin

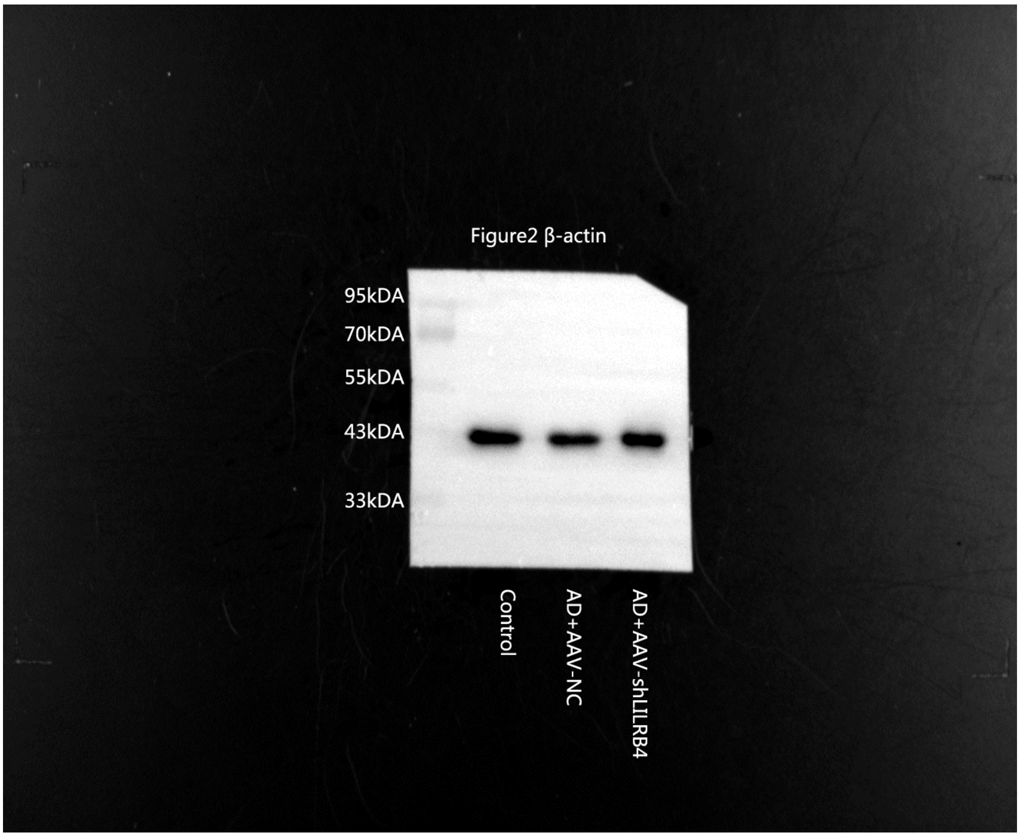

Figure3B Caspase-1(Contains multiple exposures)

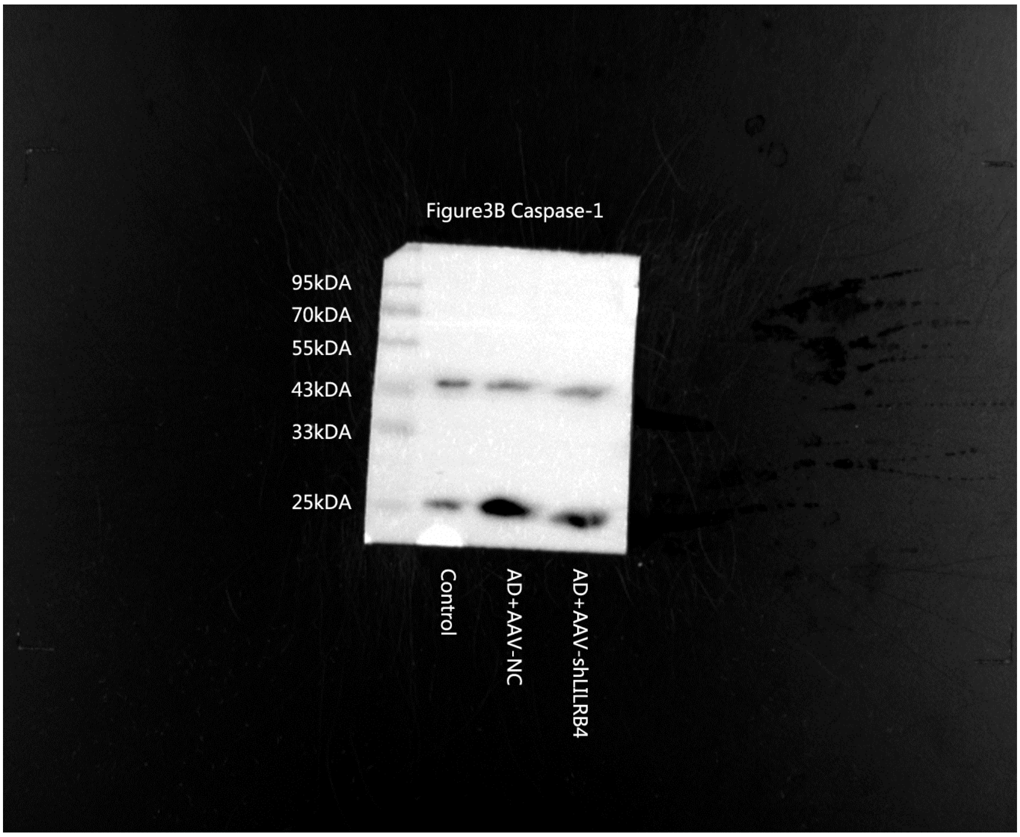

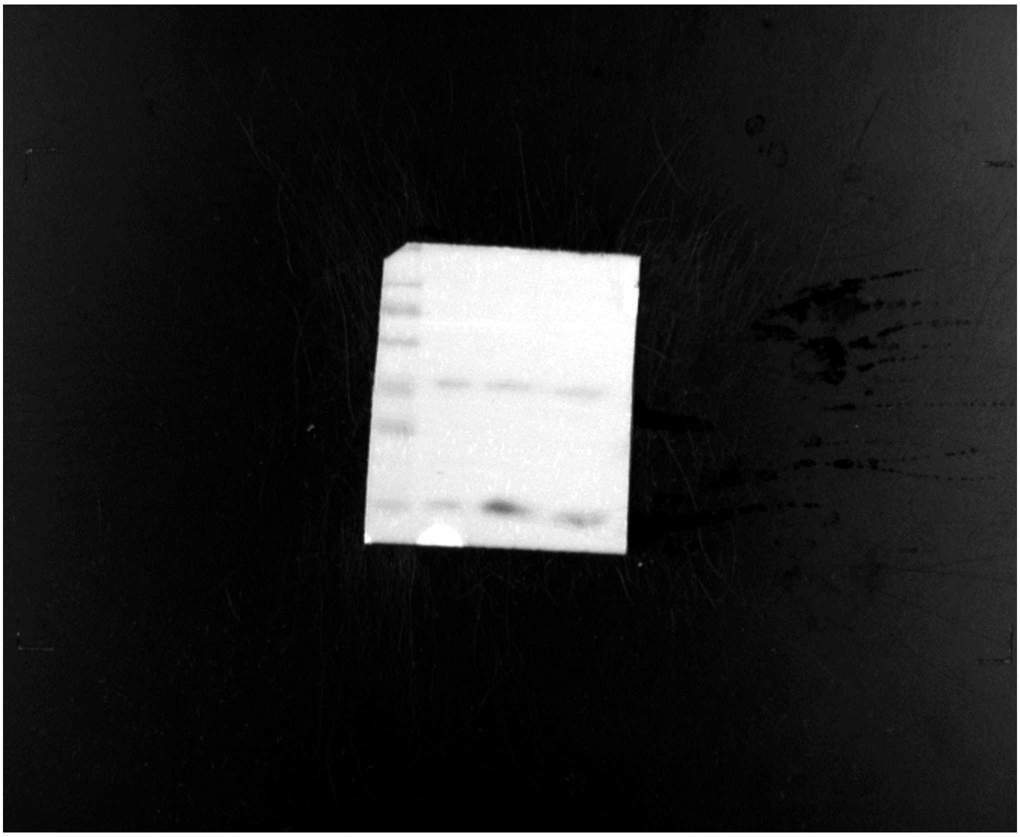

Figure3B GSDMD-N(Contains multiple exposures)

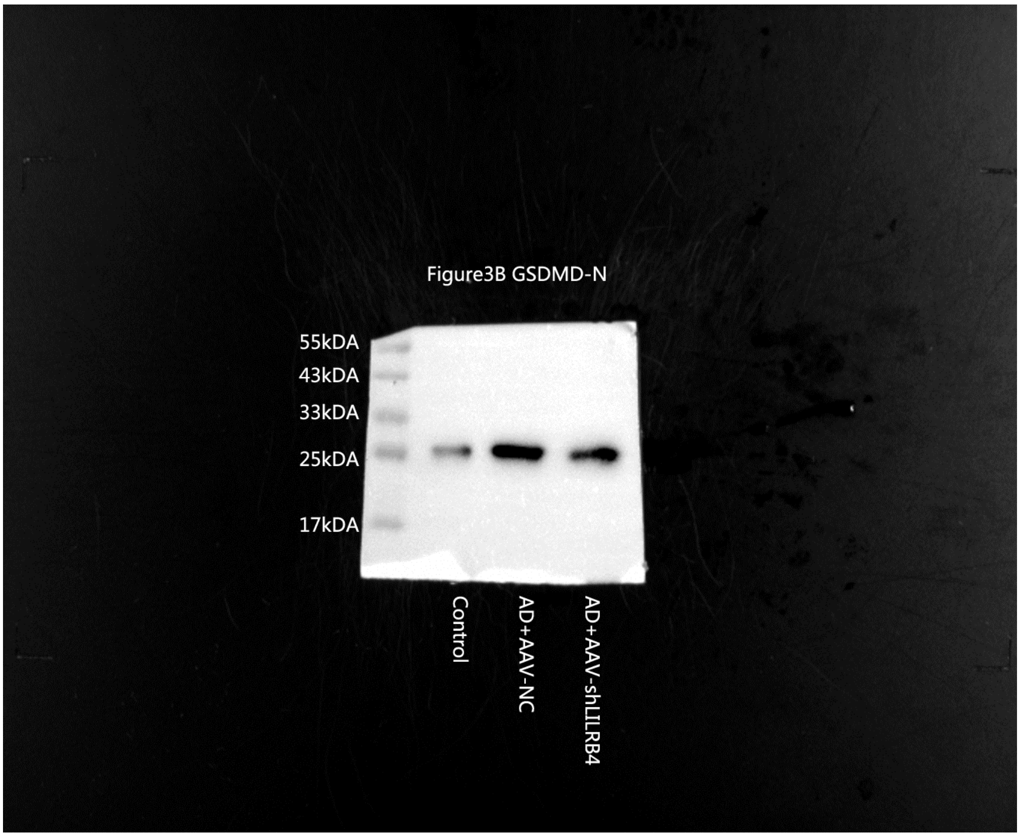

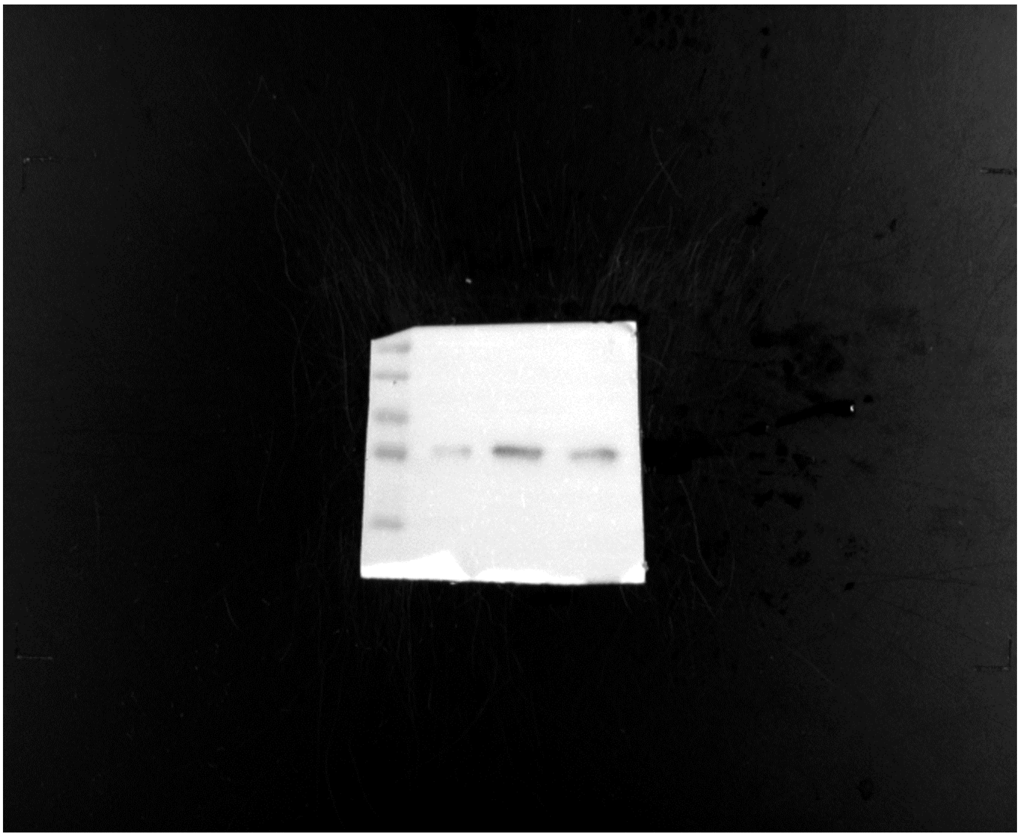

Figure3B NLRP3(Contains multiple exposures)

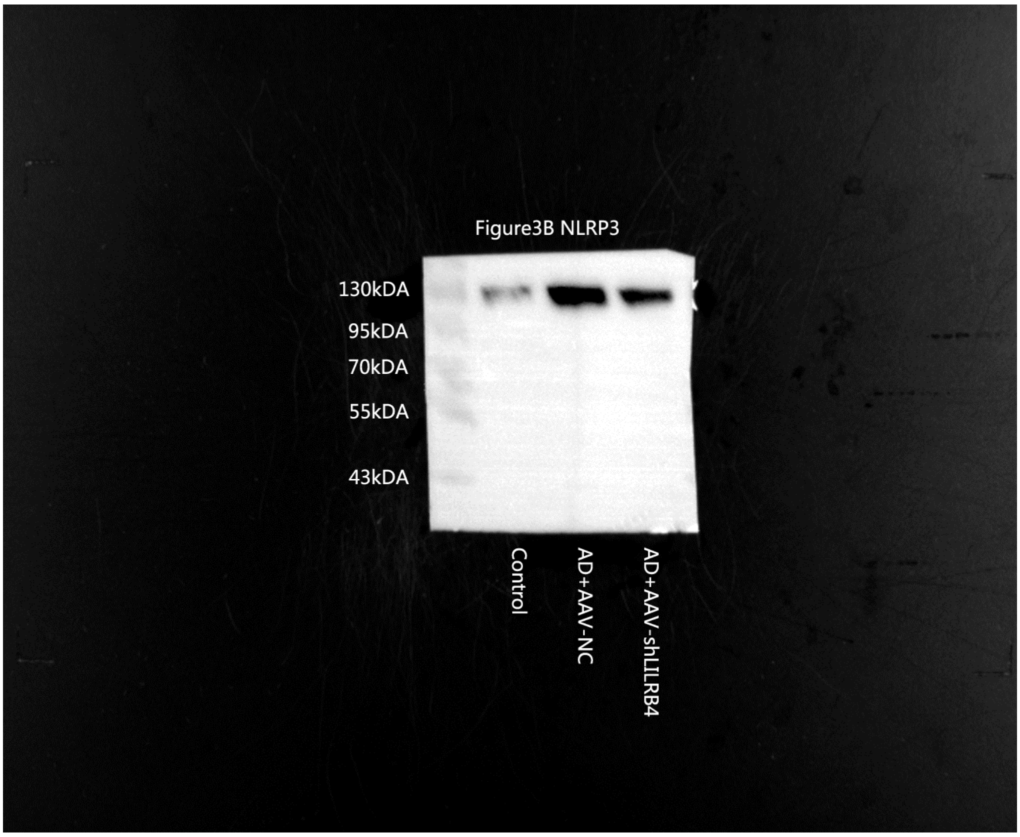

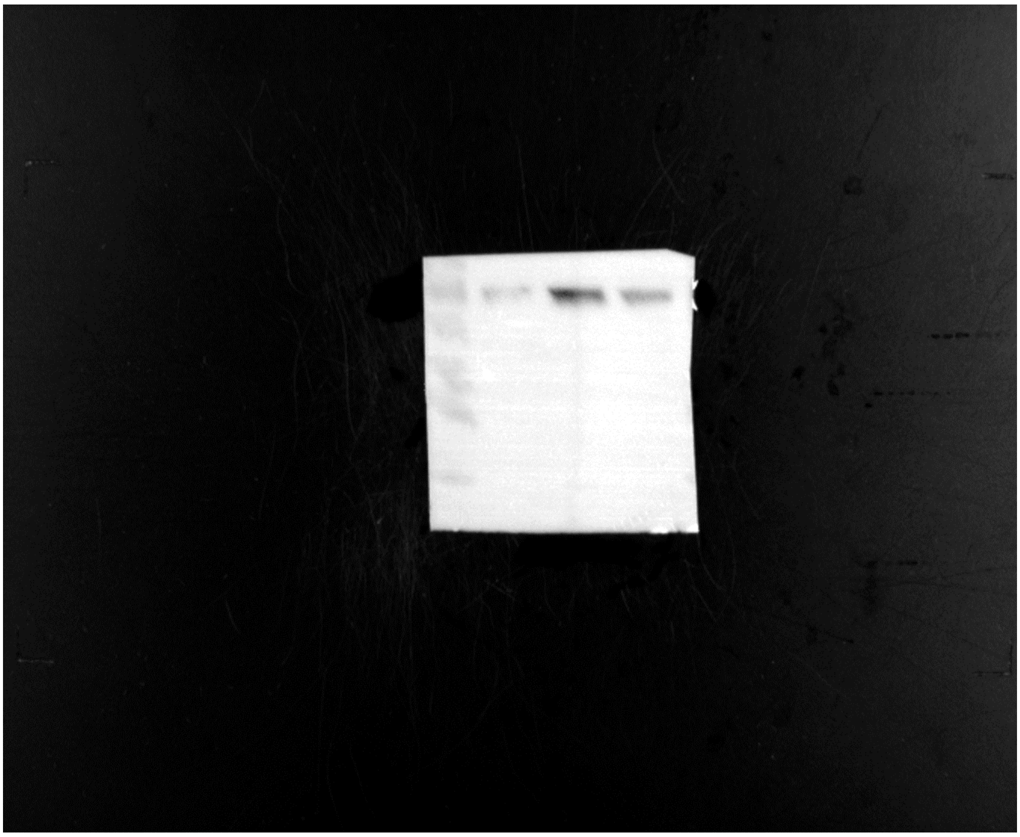

Figure3B β-actin

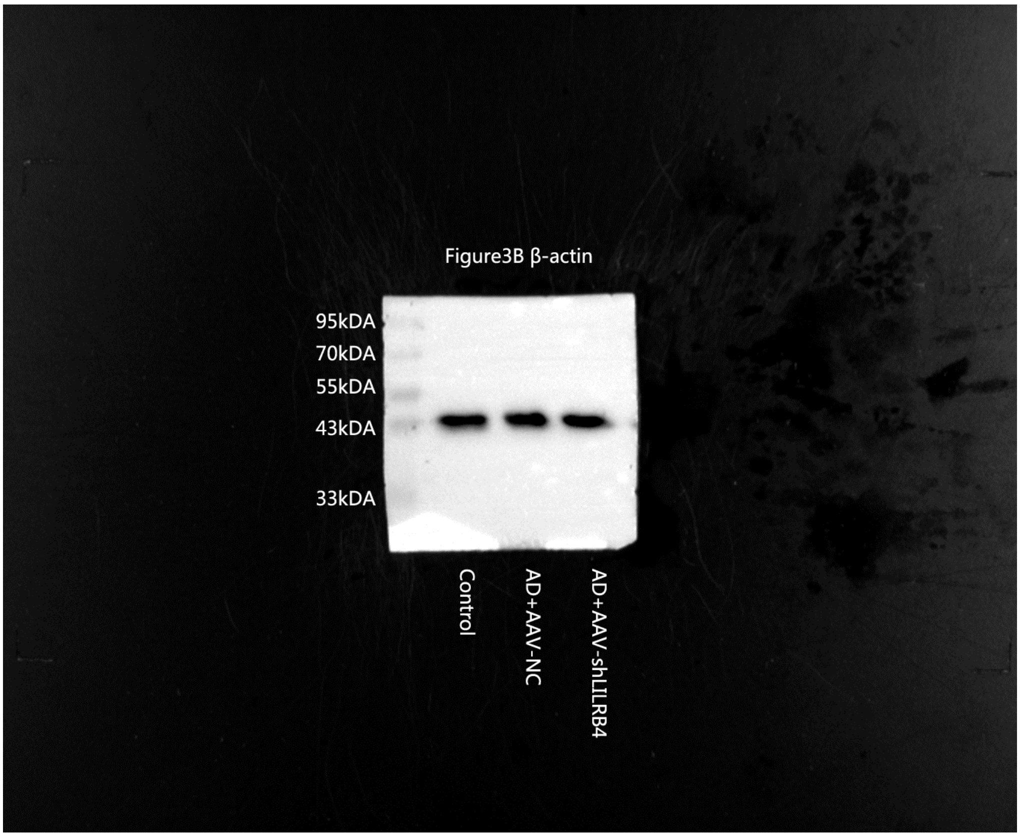

Figure4A LOX(Contains multiple exposures)

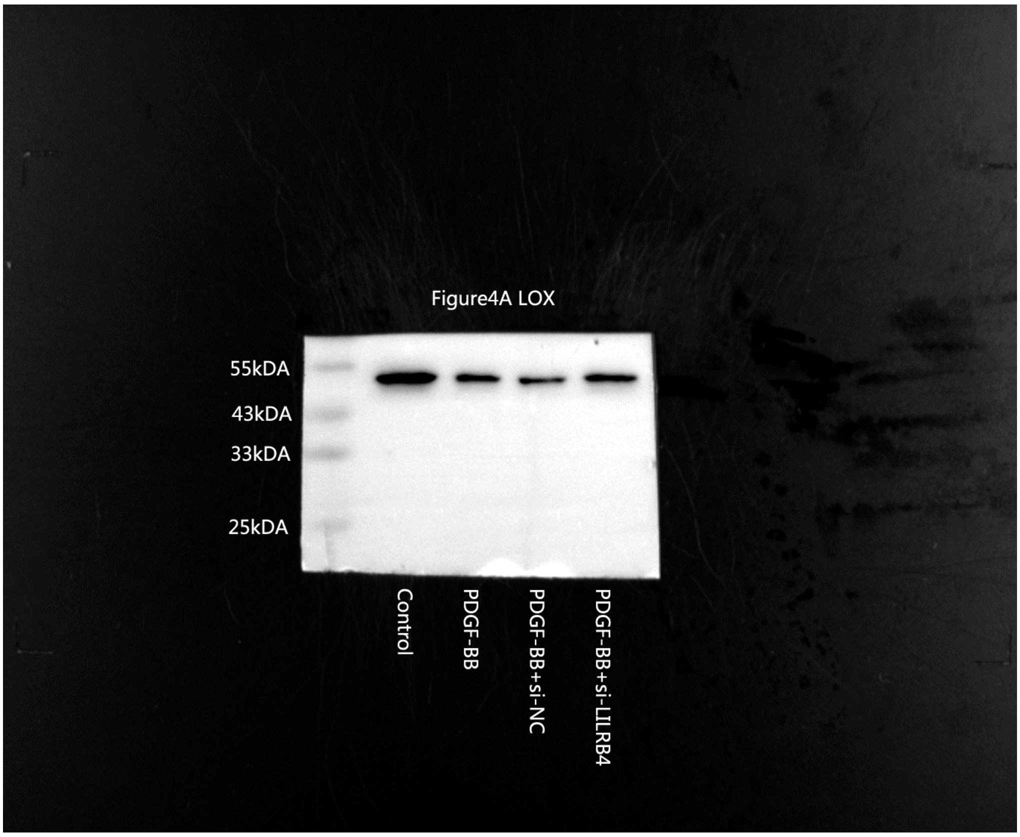

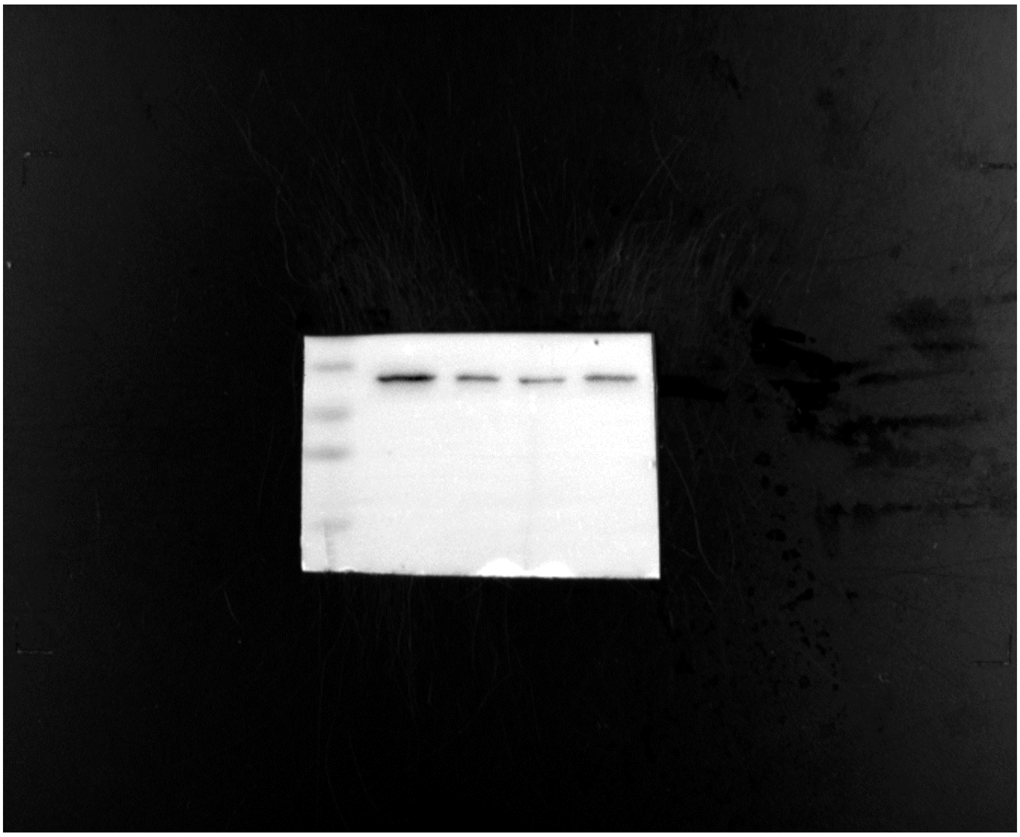

Figure4A MMP-2(Contains multiple exposures)

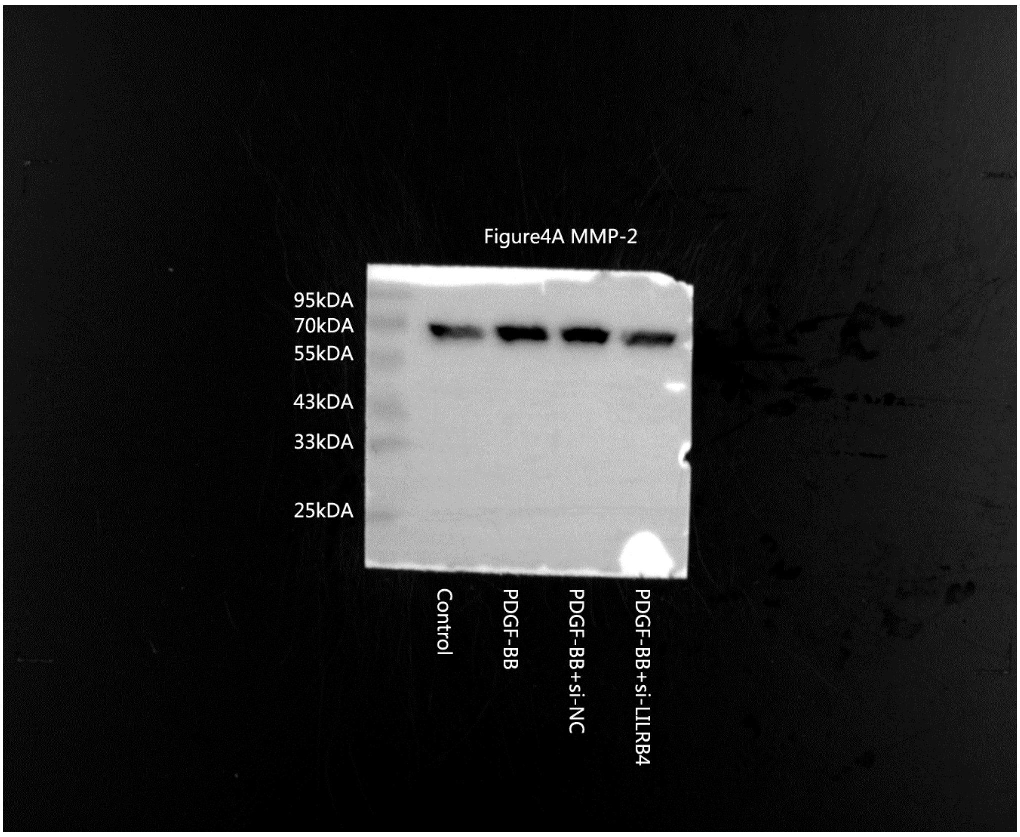

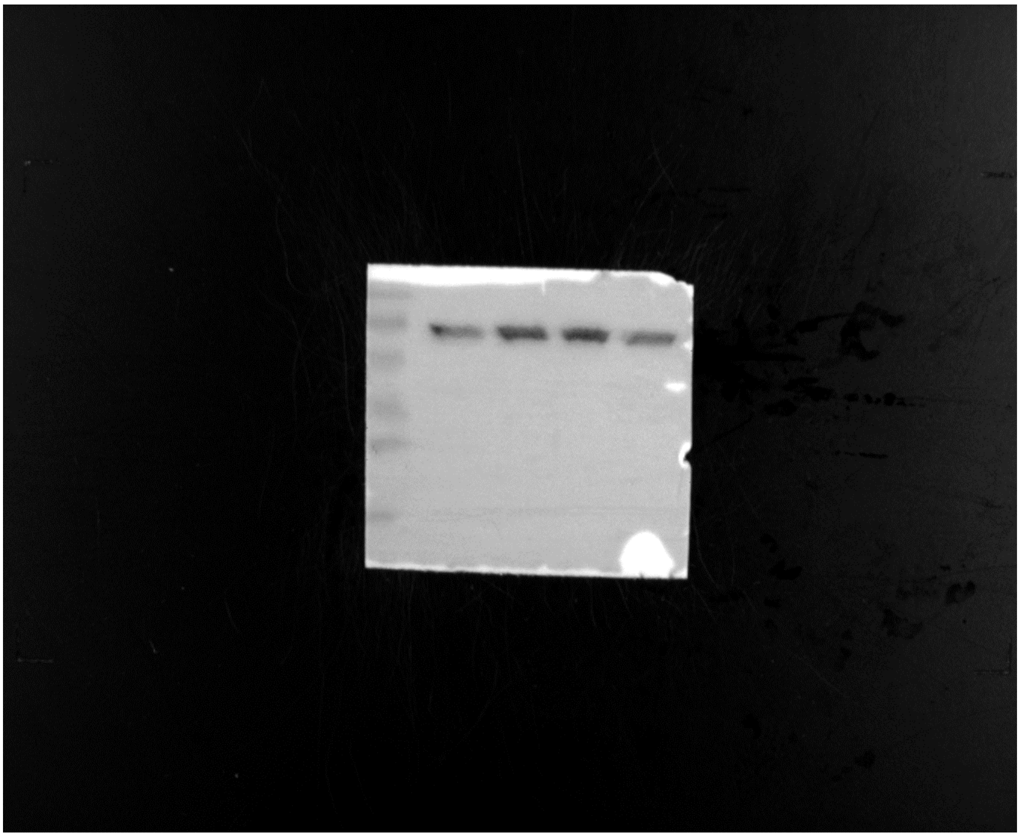

Figure4A MMP-9(Contains multiple exposures)

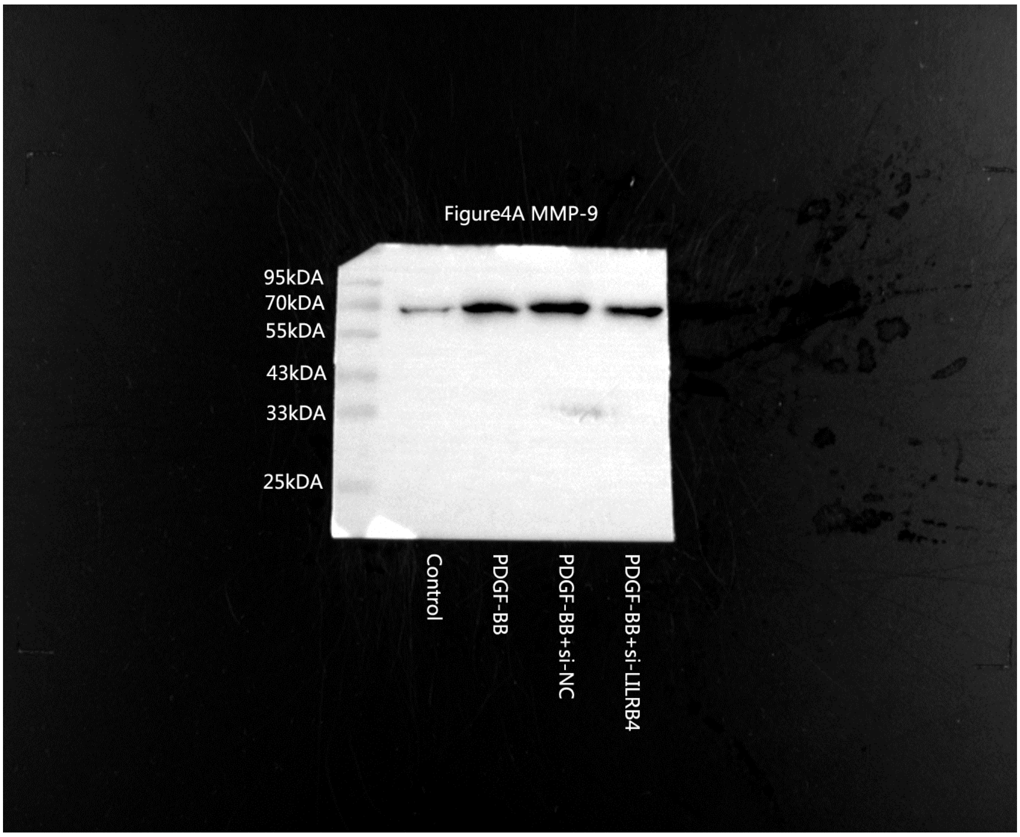

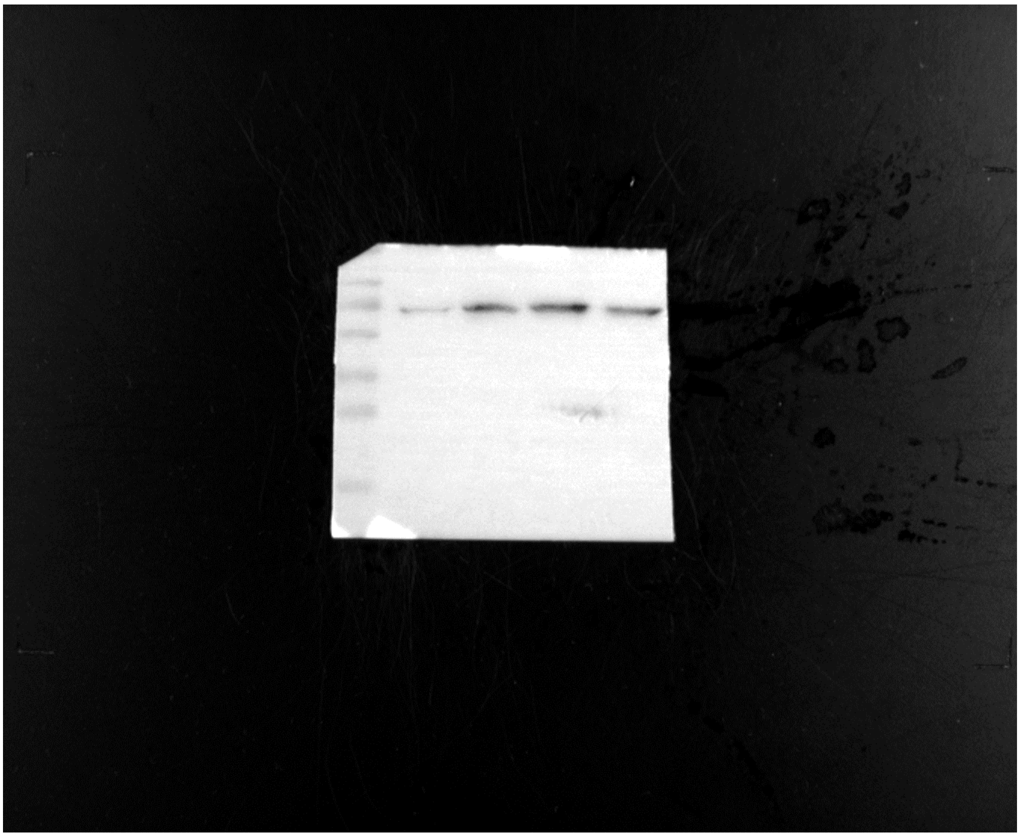

Figure4A S100A4(Contains multiple exposures)

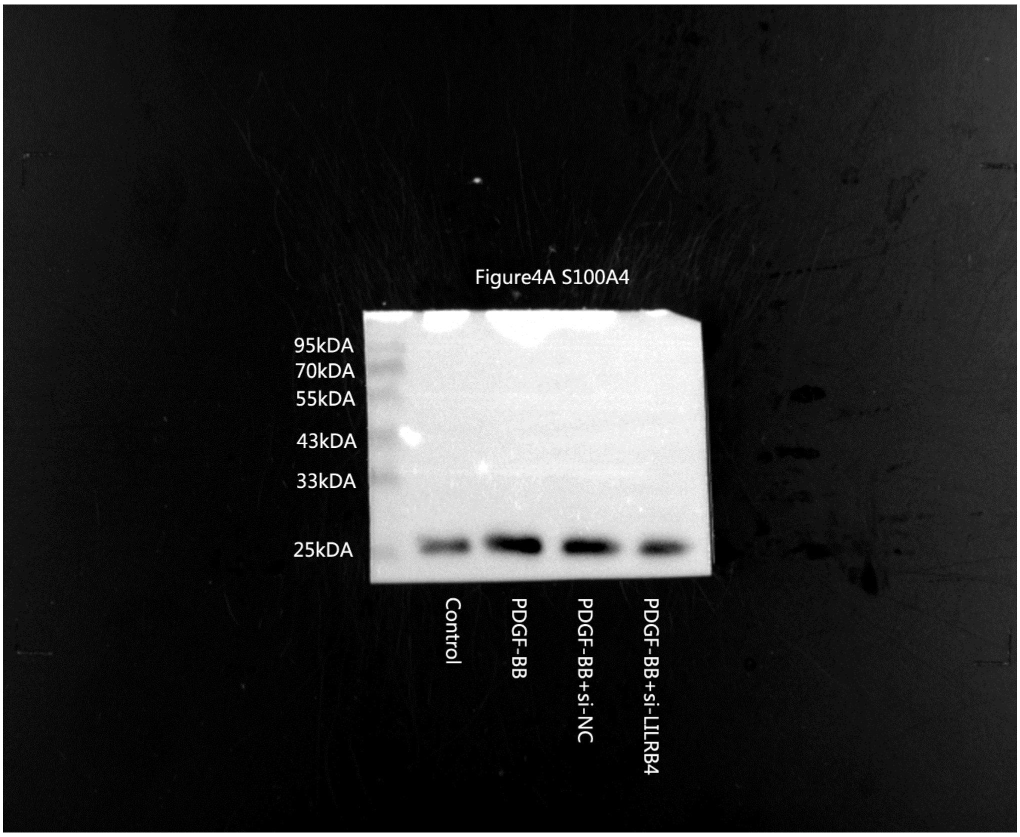

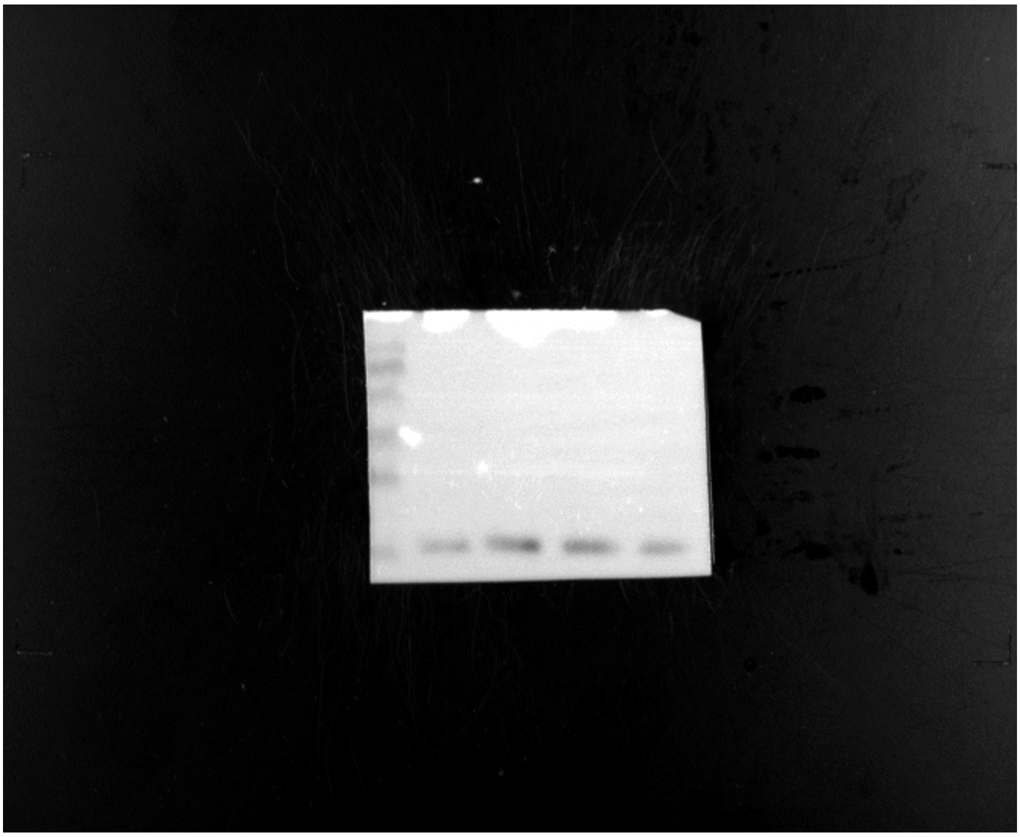

Figure4A SM22-α

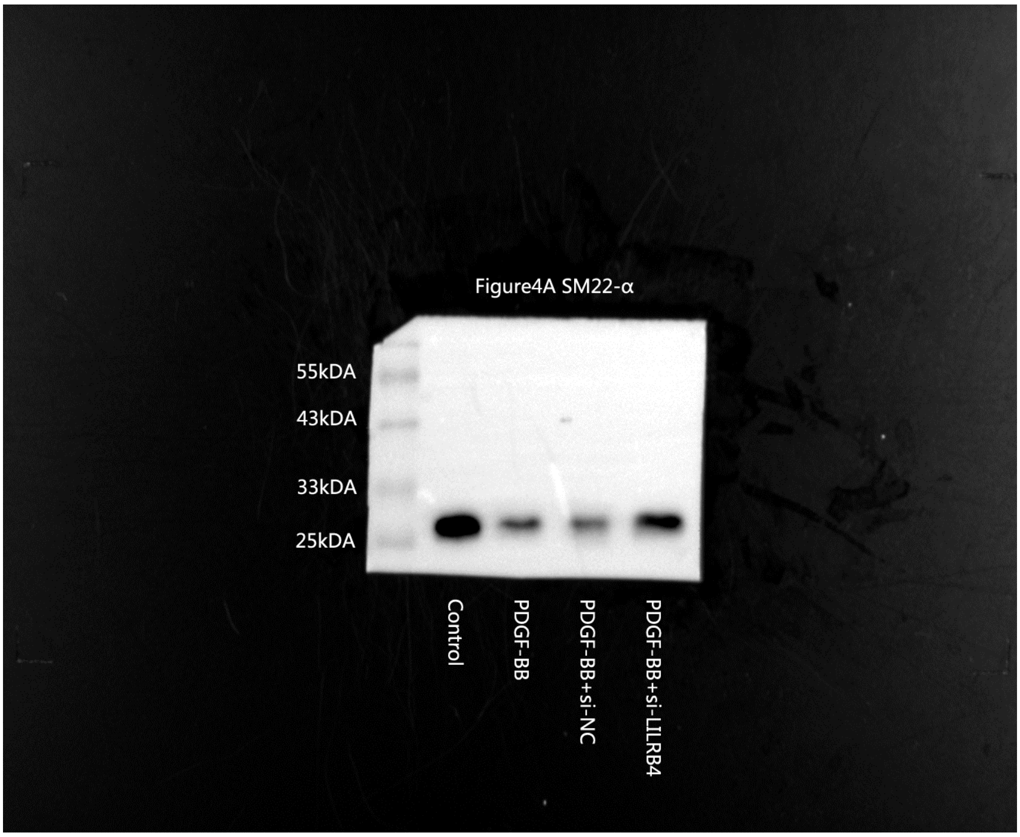

Figure4A α-SMA

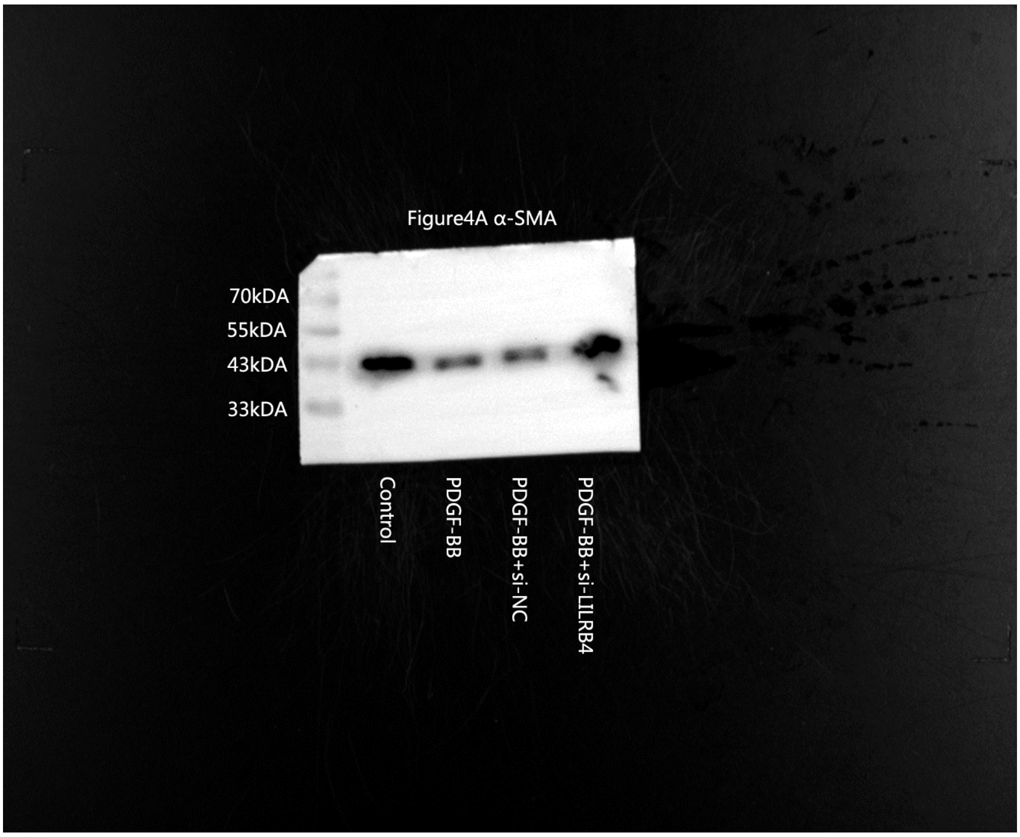

Figure4A β-actin(Contains multiple exposures)

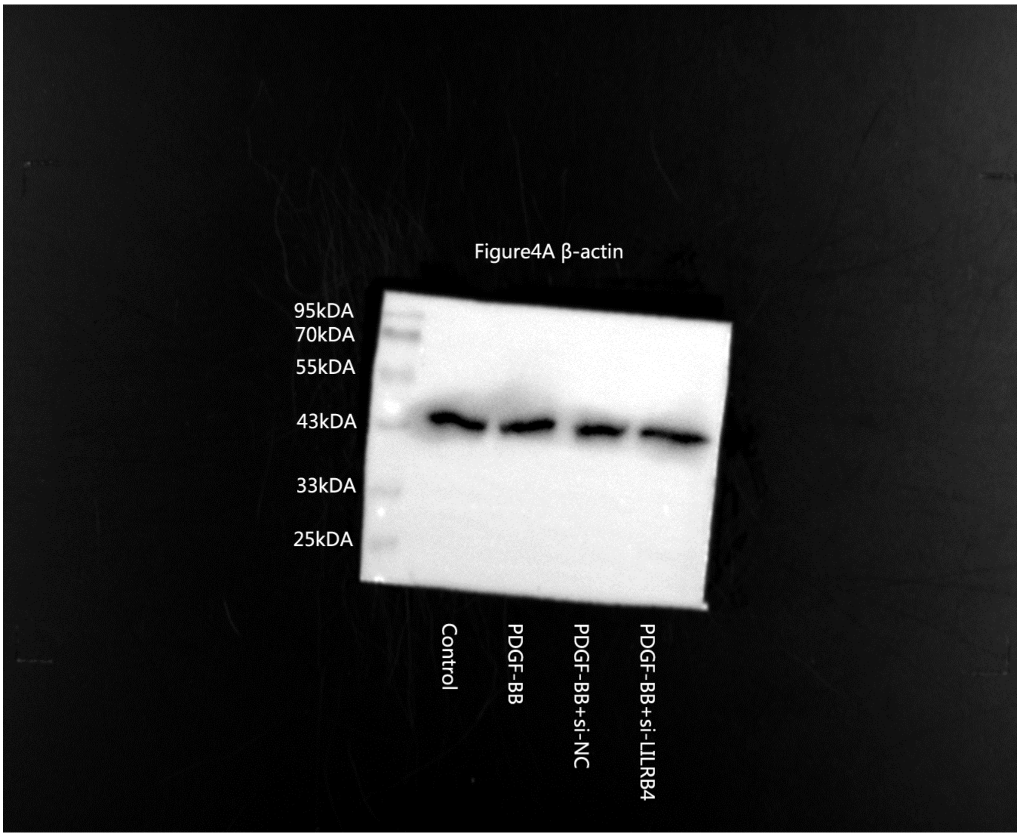

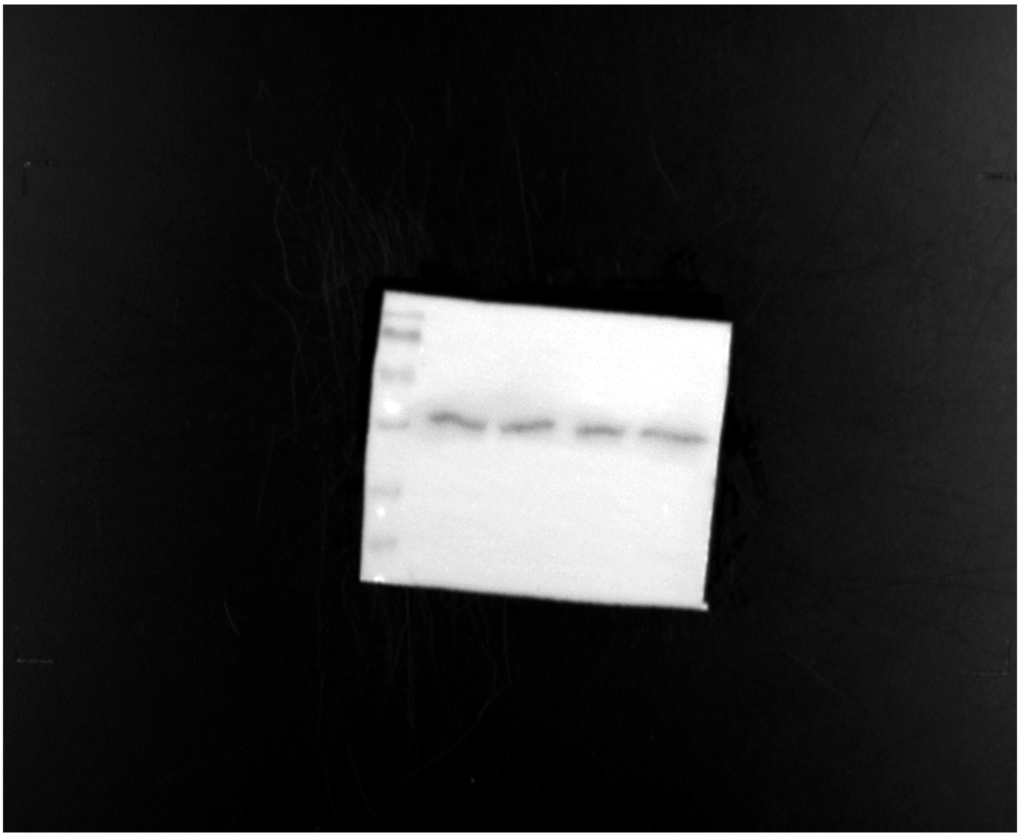

Figure5C JAK2

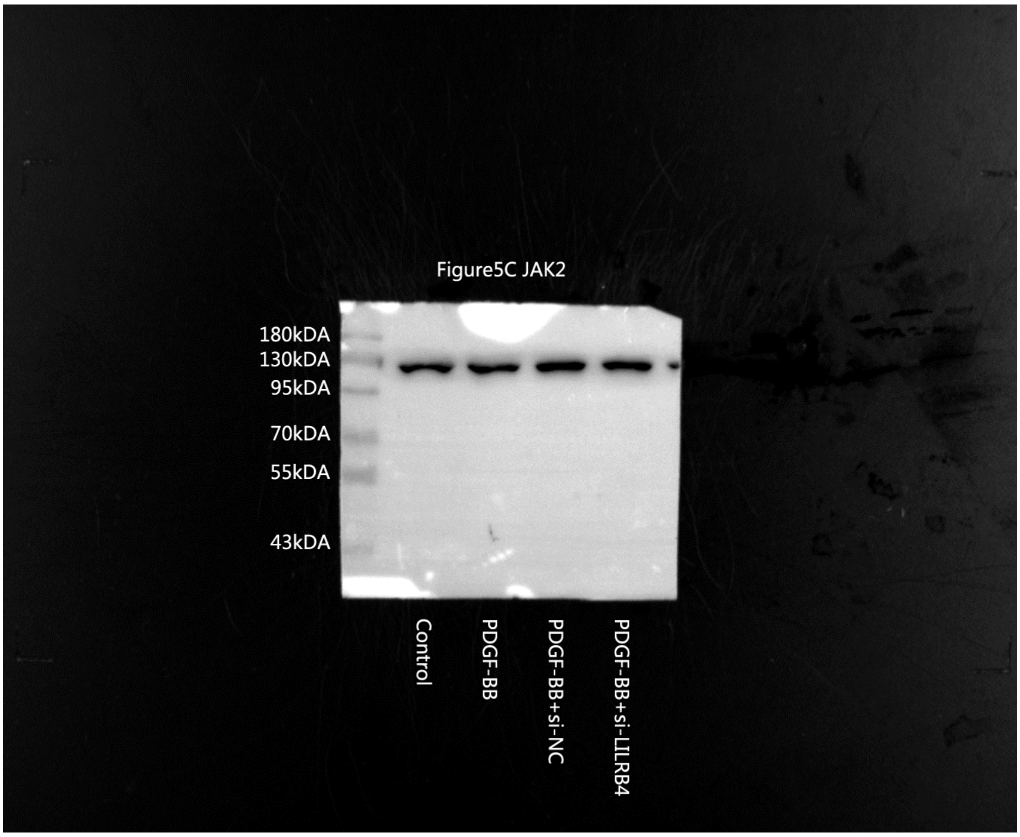

Figure5C p-JAK2(Contains multiple exposures)

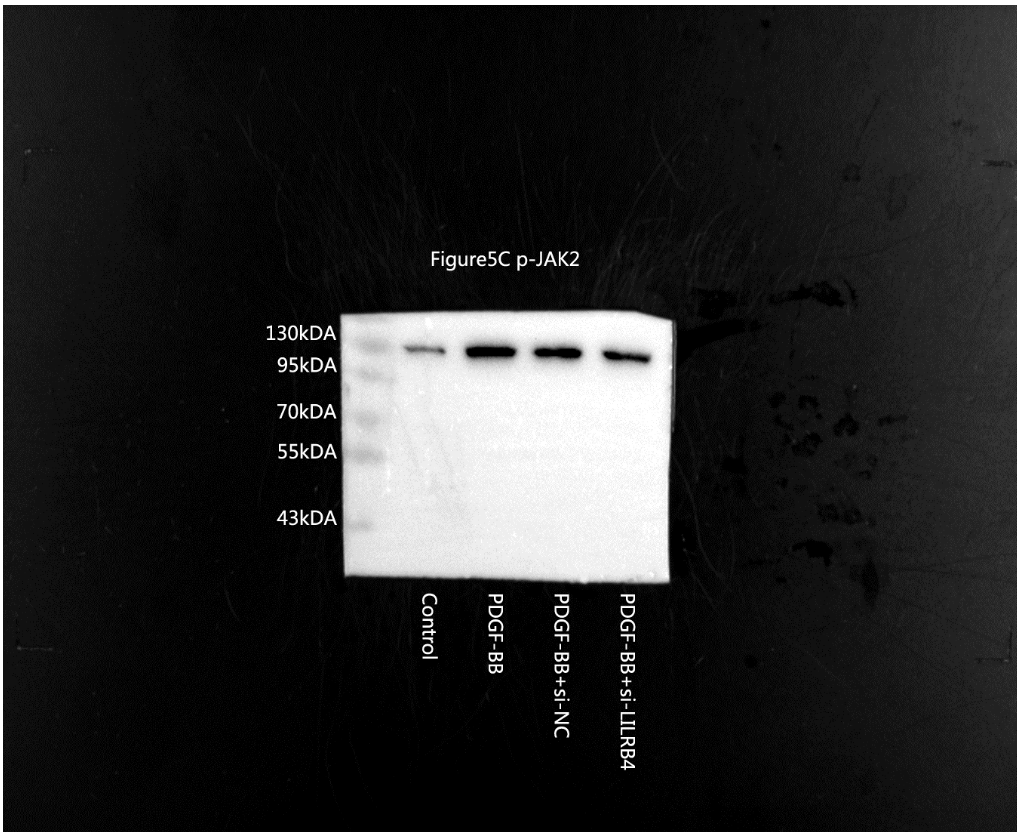

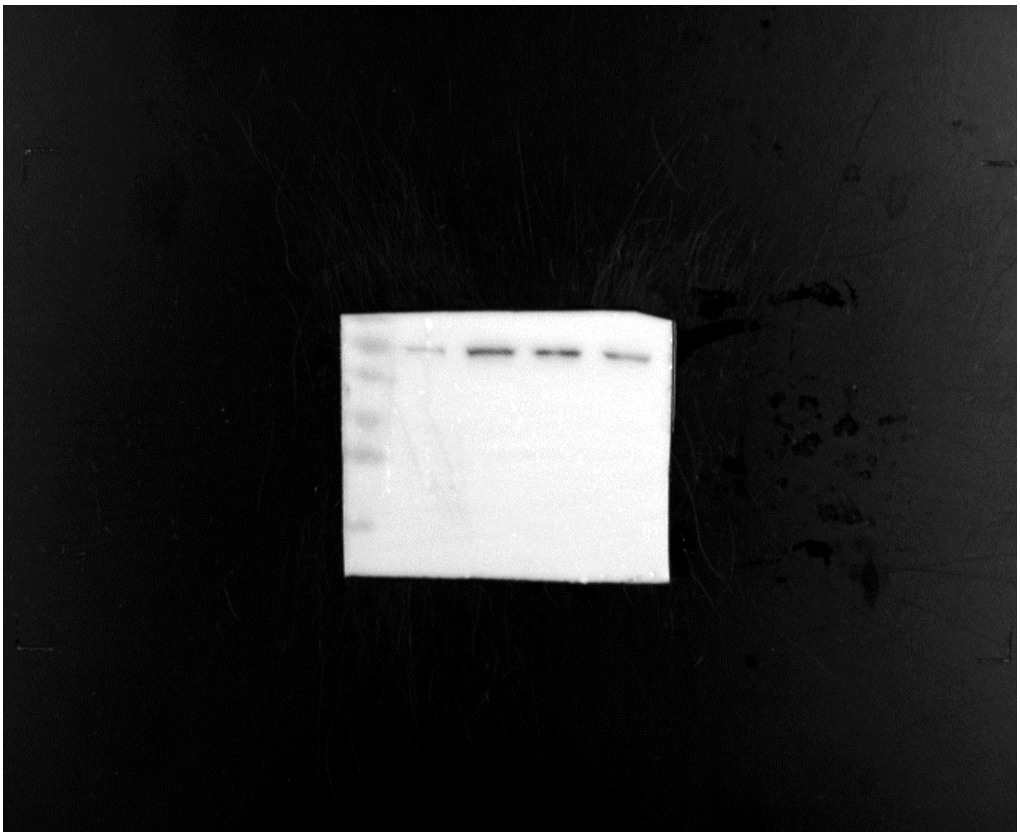

Figure5C p-STAT3

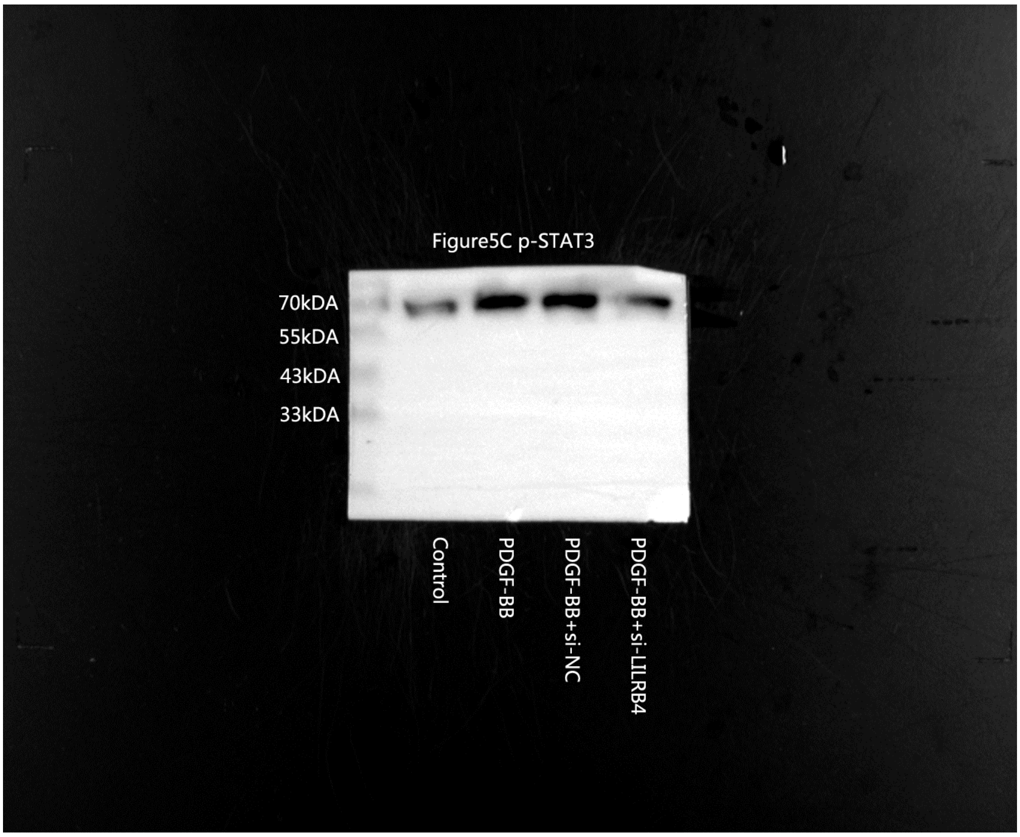

Figure5C STAT3(Contains multiple exposures)

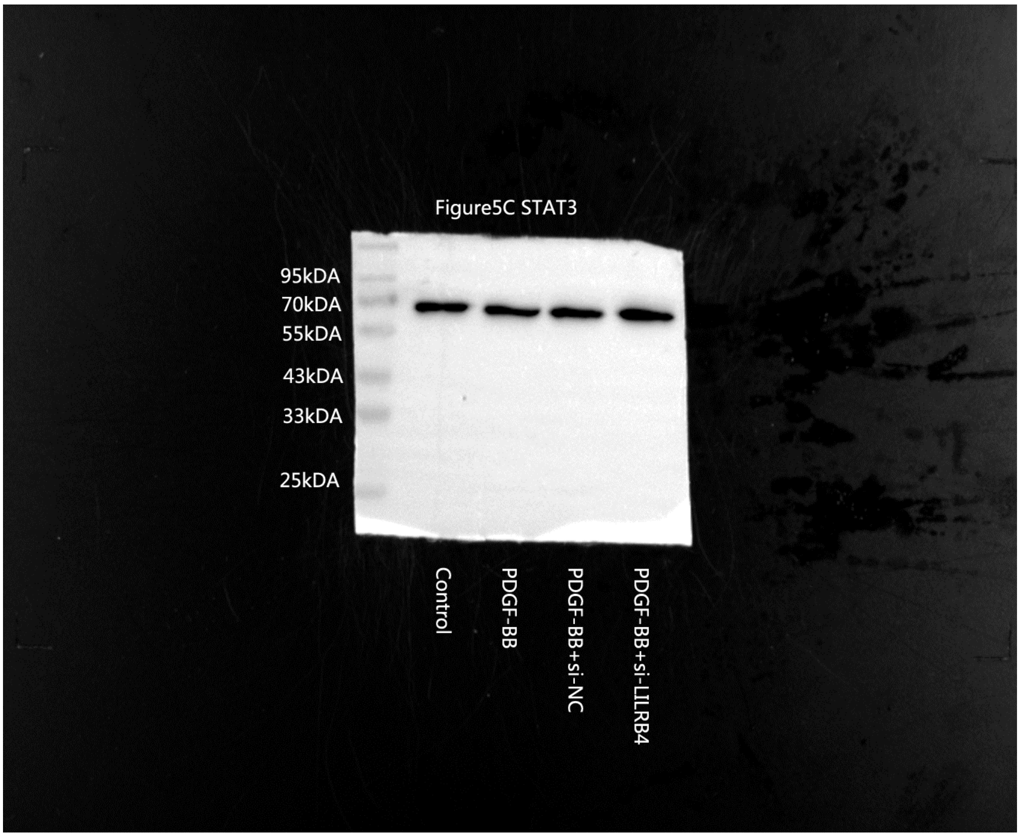

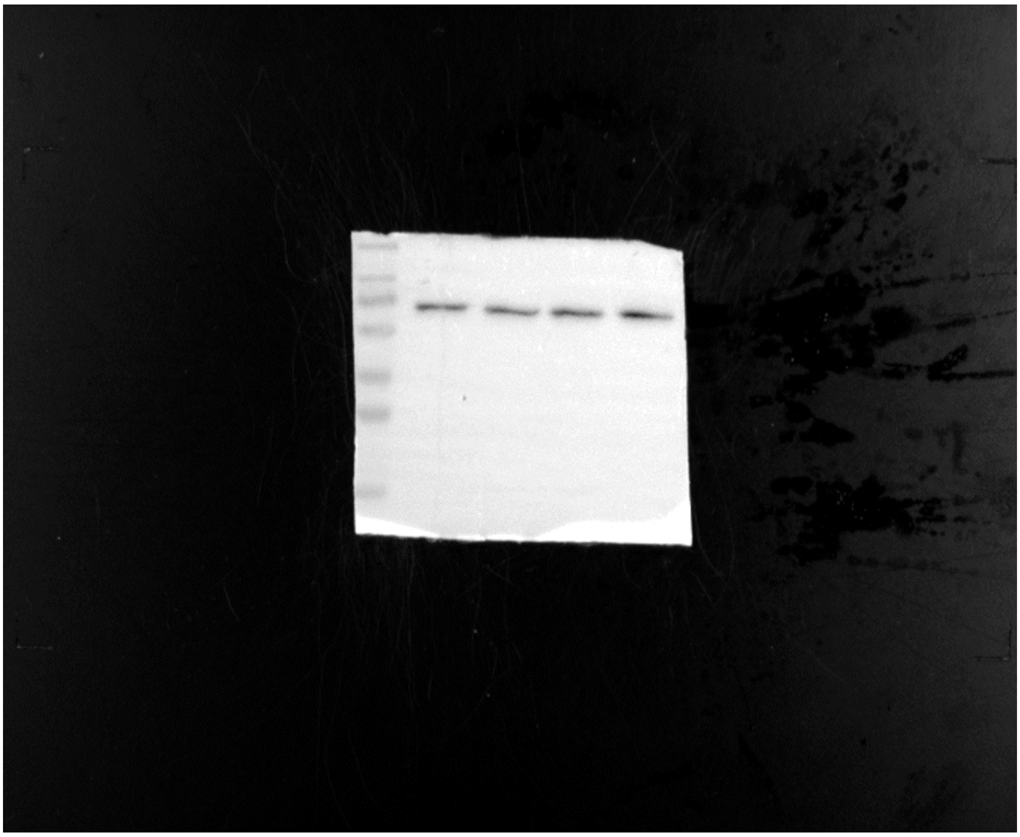

Figure5C β-actin

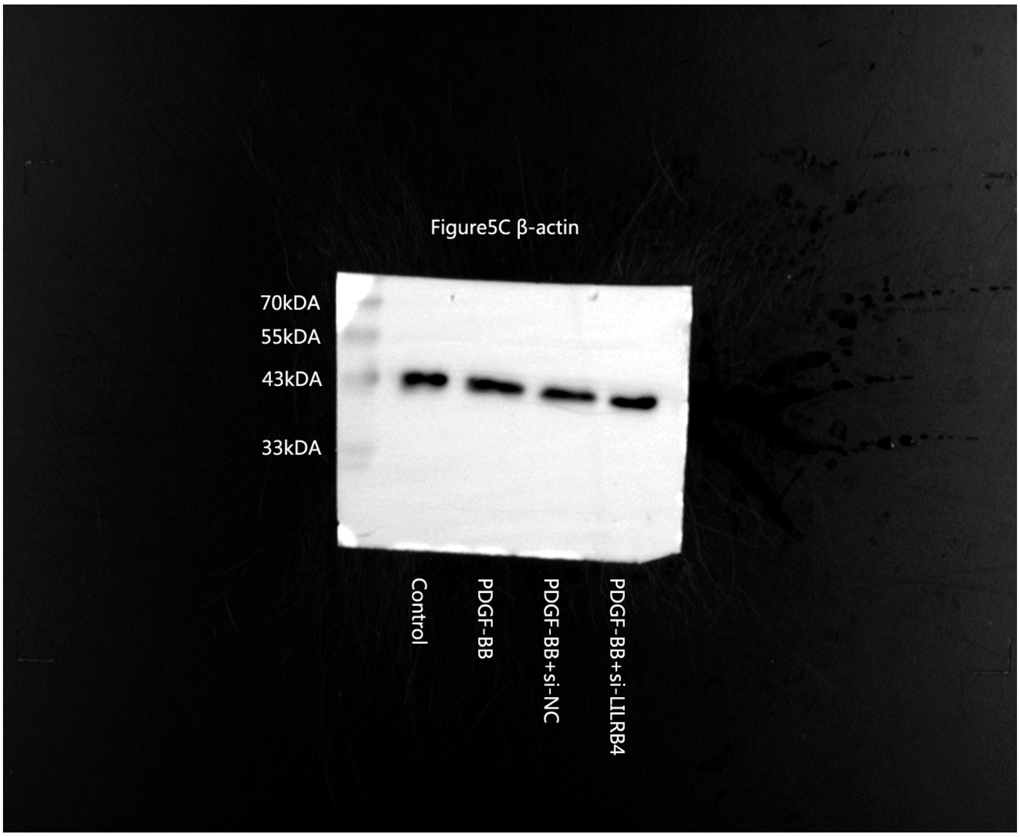

Figure5D p-JAK2

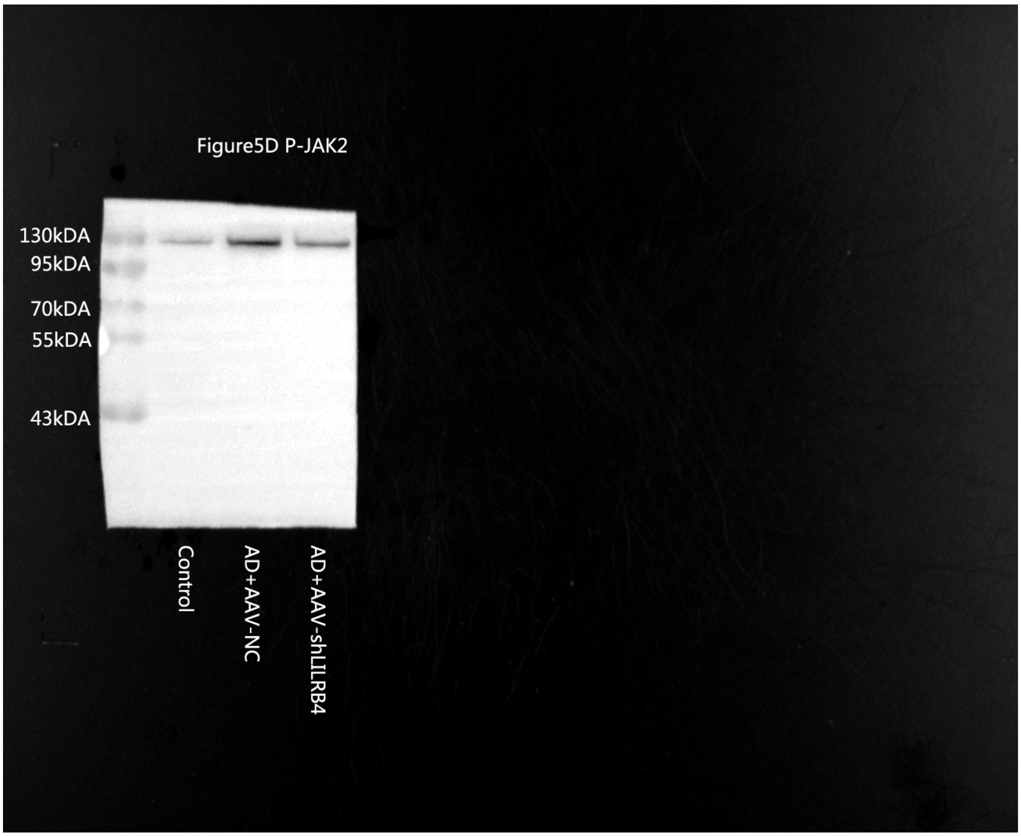

Figure5D JAK2

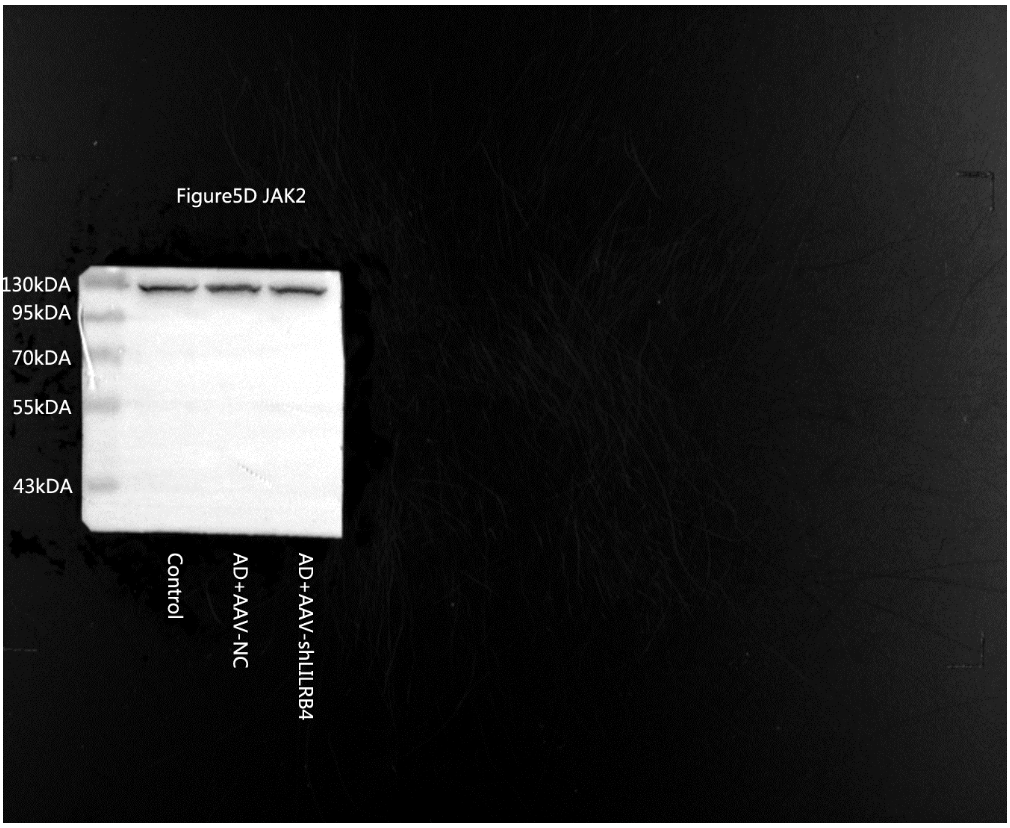

Figure5D p-STAT3

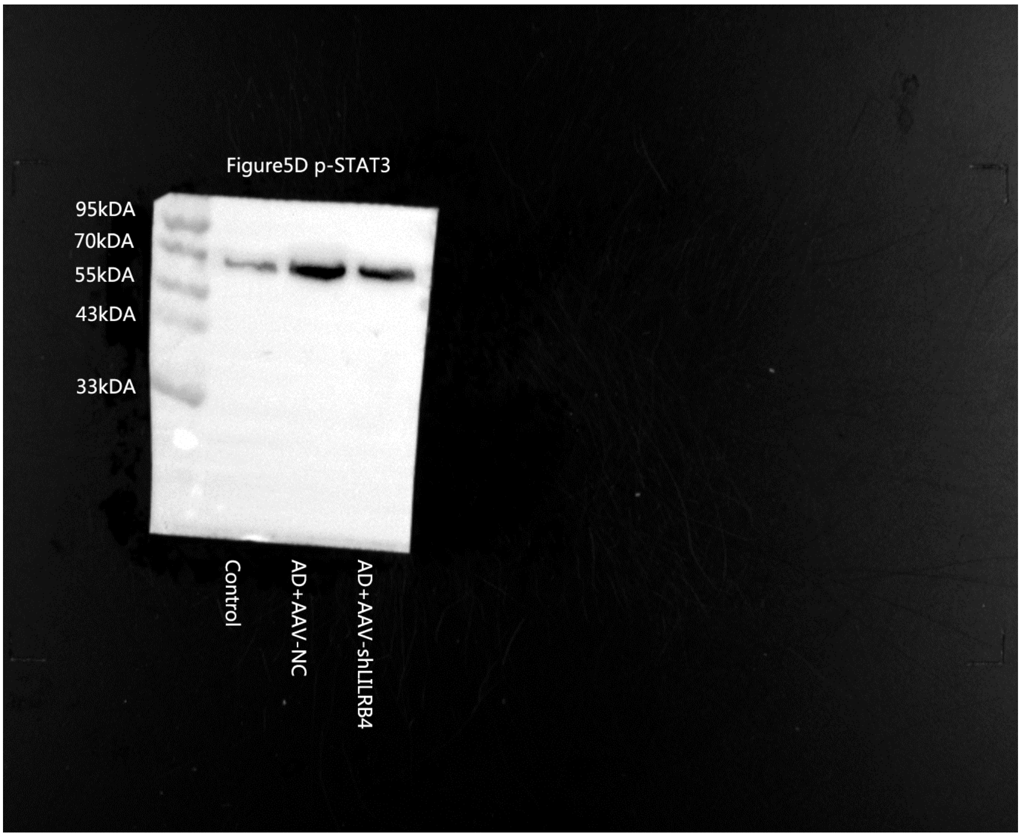

Figure5D STAT3

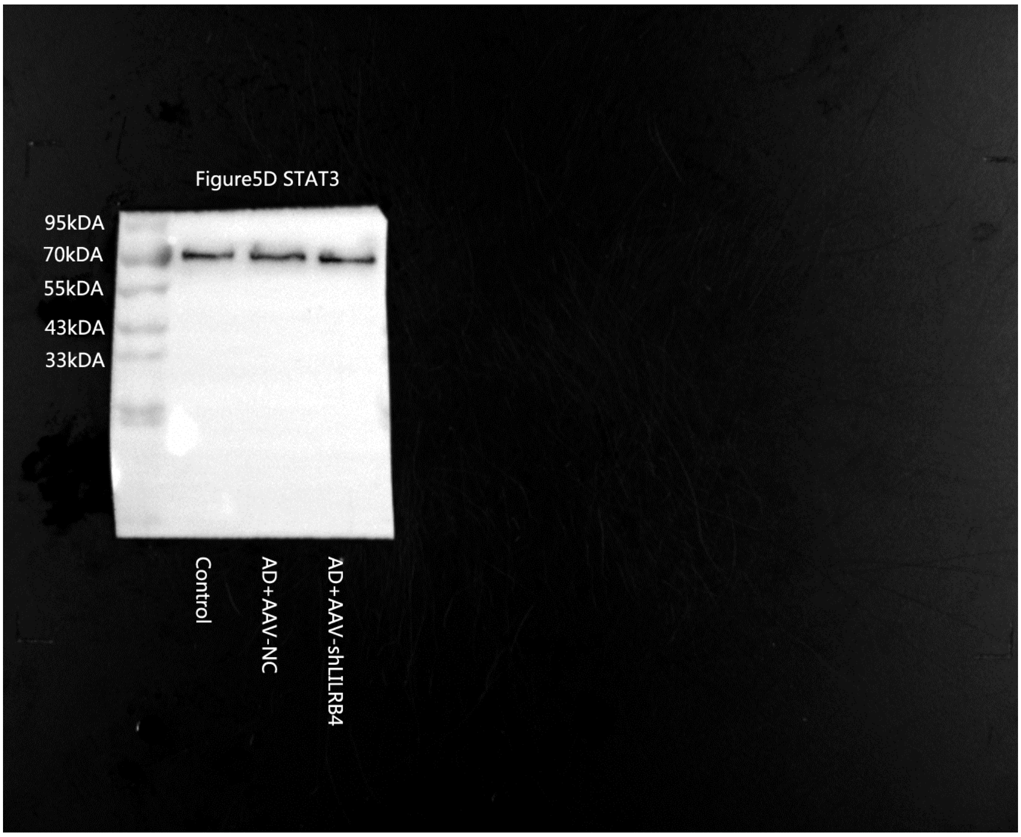

Figure5D β-actin

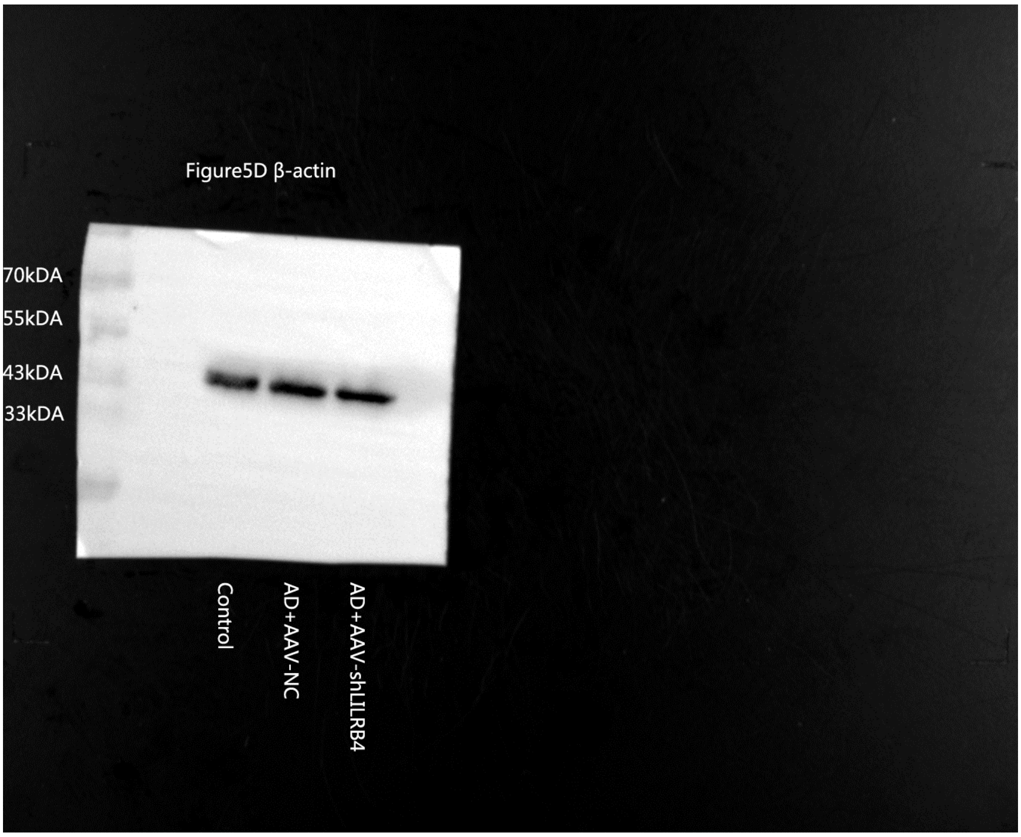

Figure7 LOX

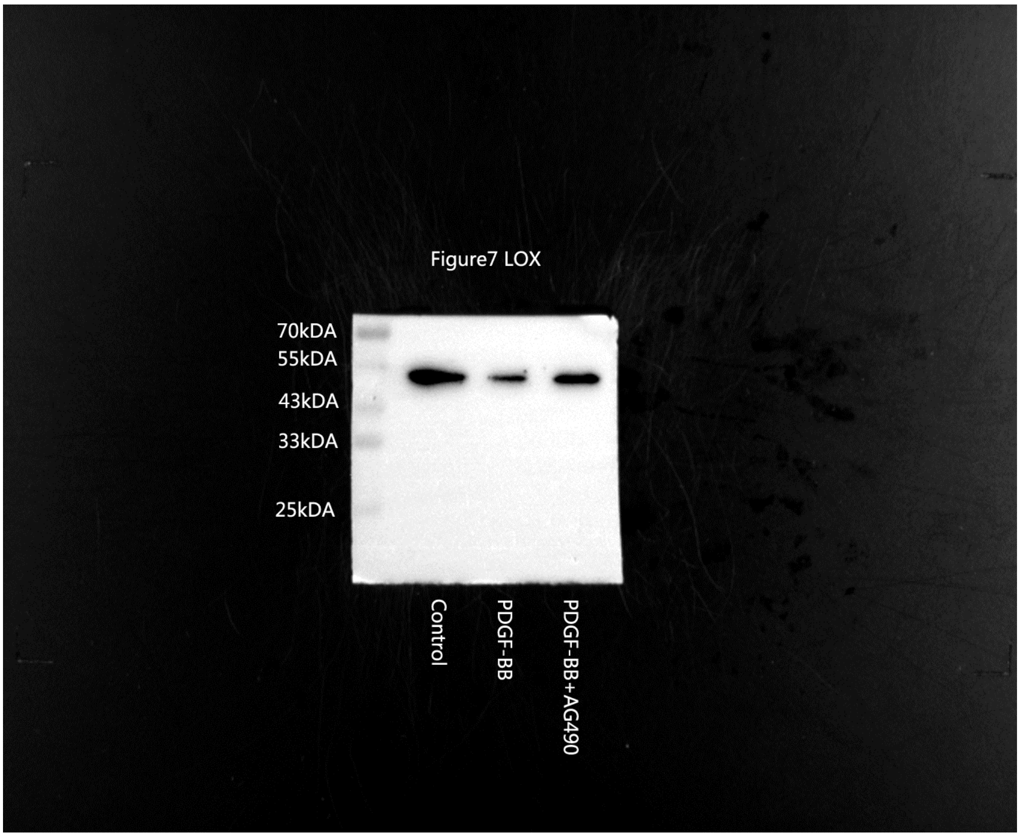

Figure7 MMP-2(Contains multiple exposures)

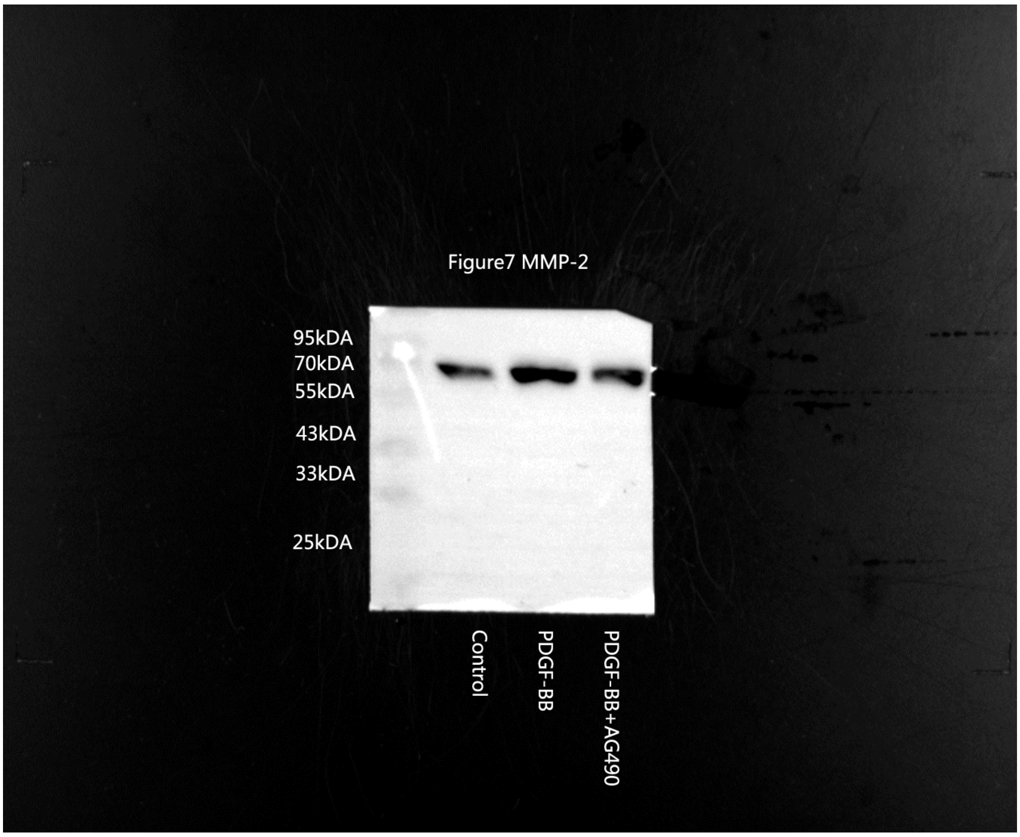

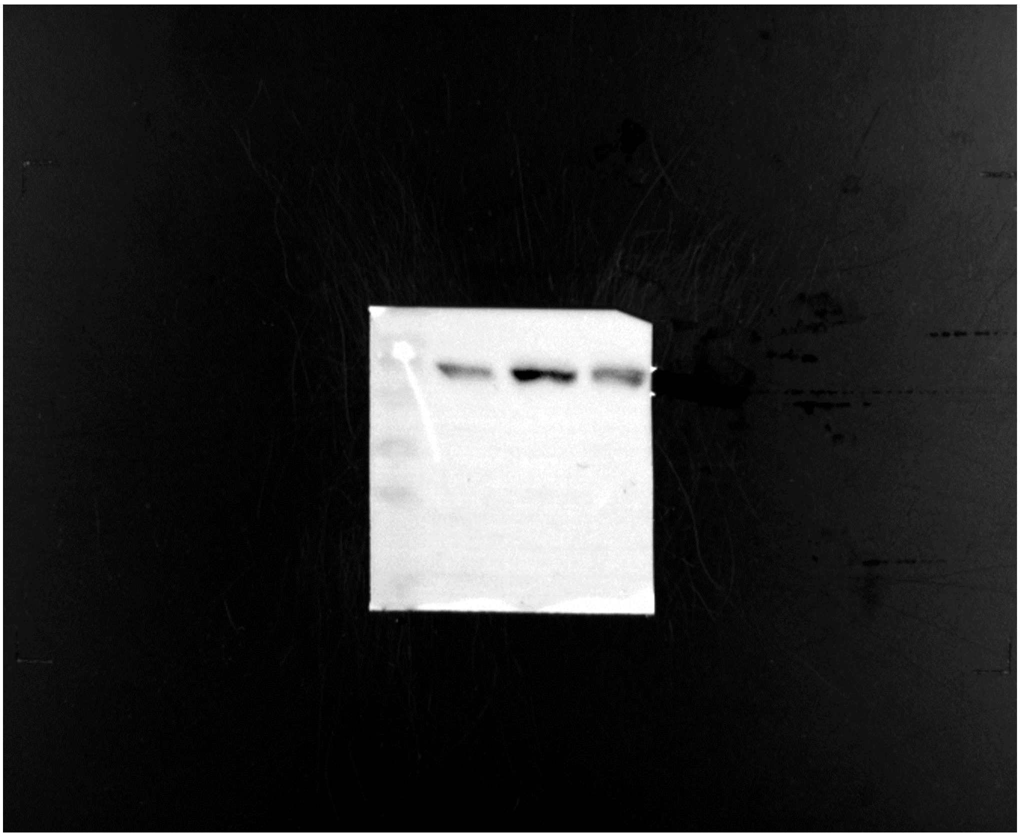

Figure7 MMP-9

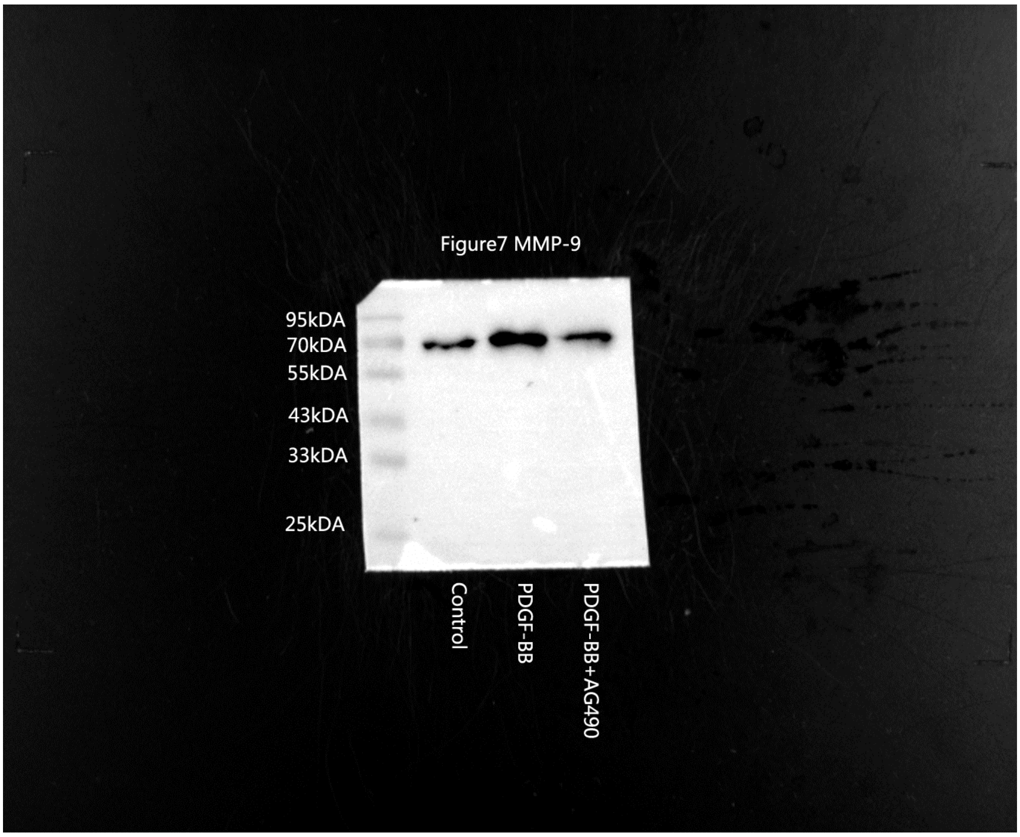

Figure7 S100A4(Contains multiple exposures)

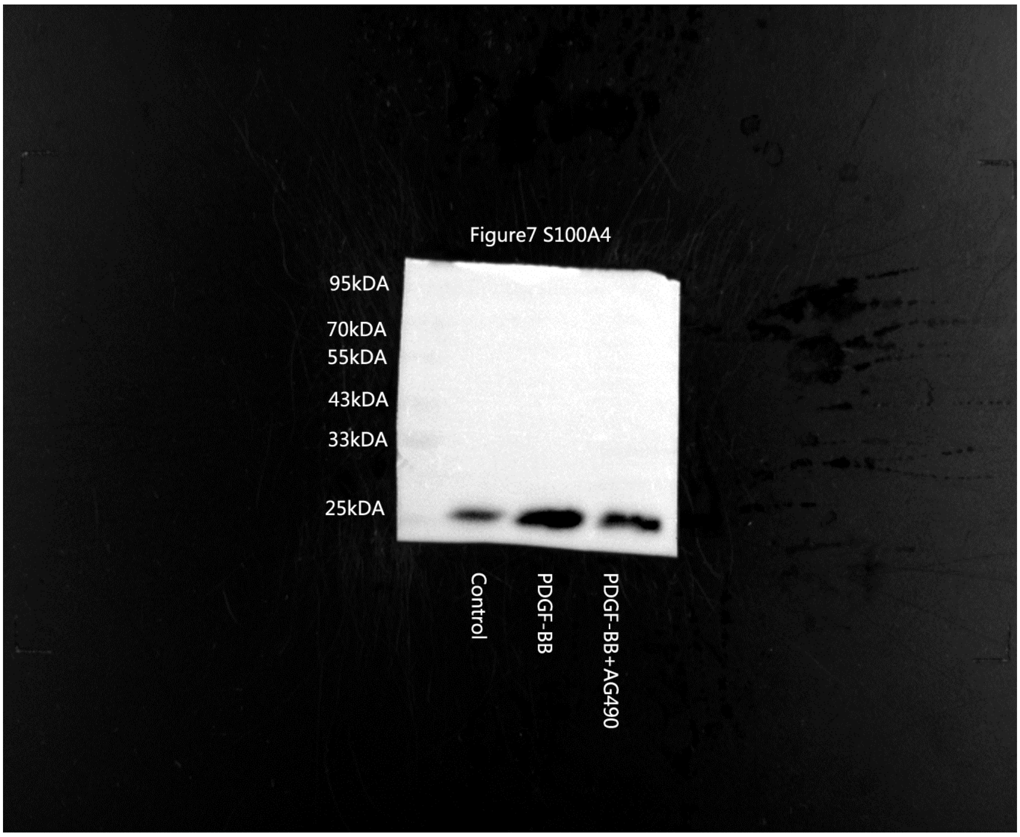

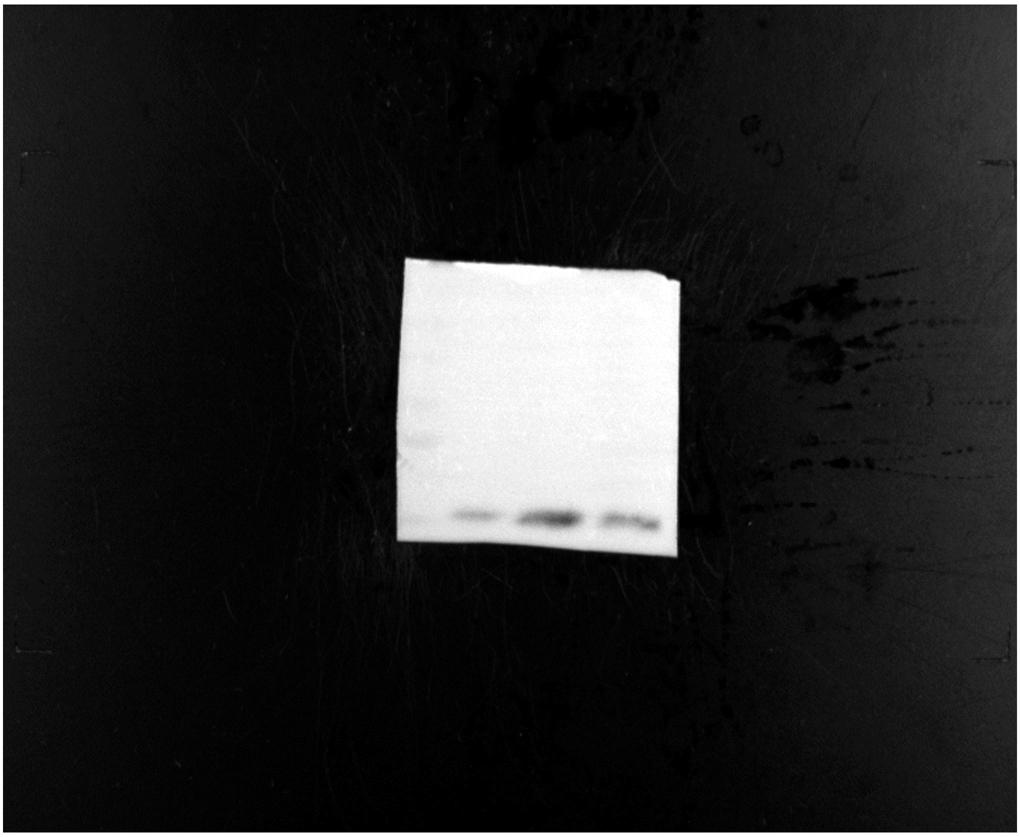

Figure7 Sm22α

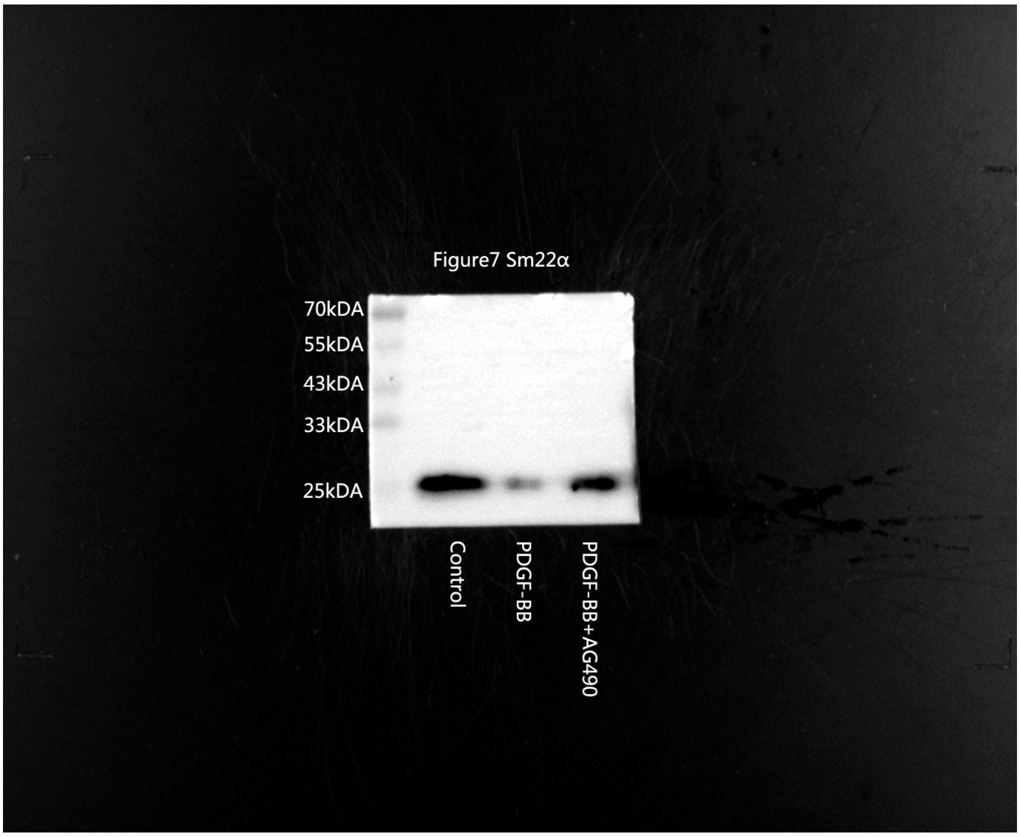

Figure7 α-SMA(Contains multiple exposures)

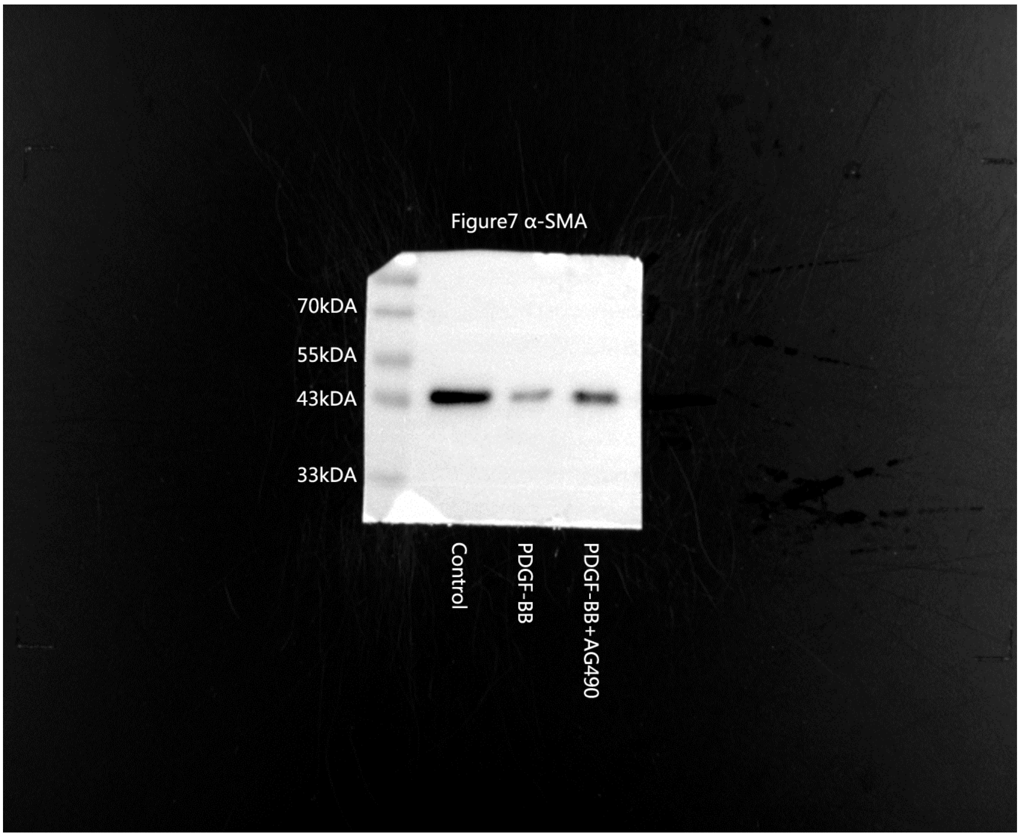

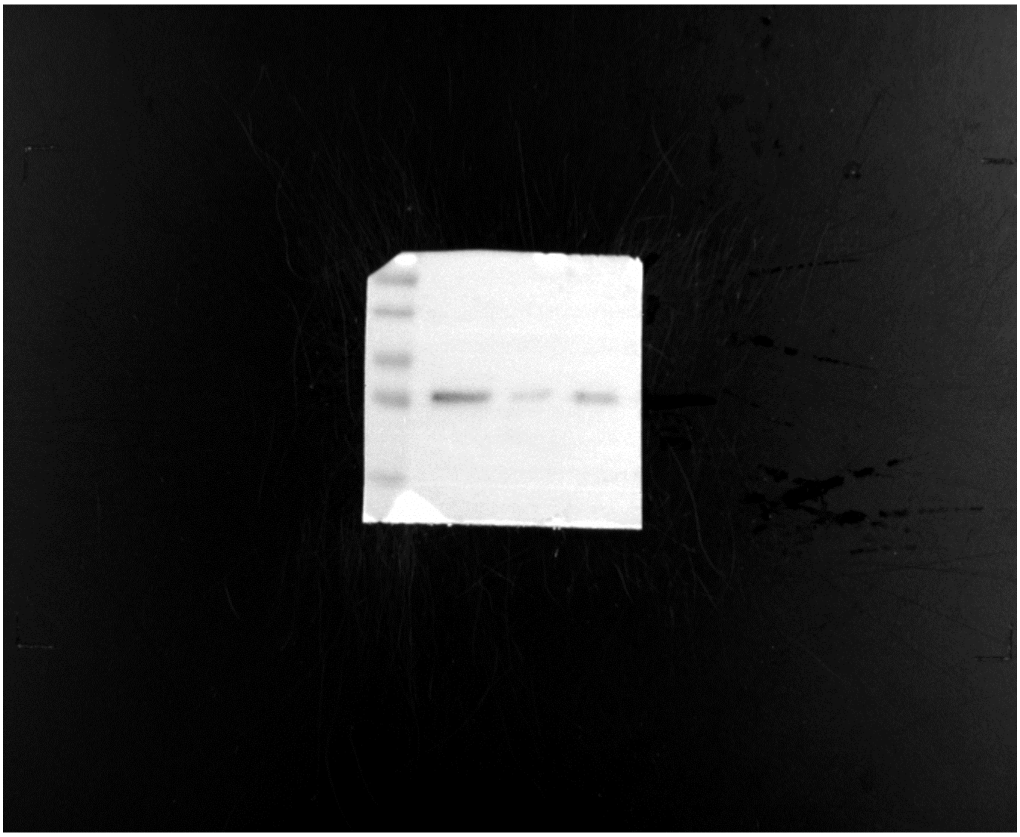

Figure7 β-actin(Contains multiple exposures)

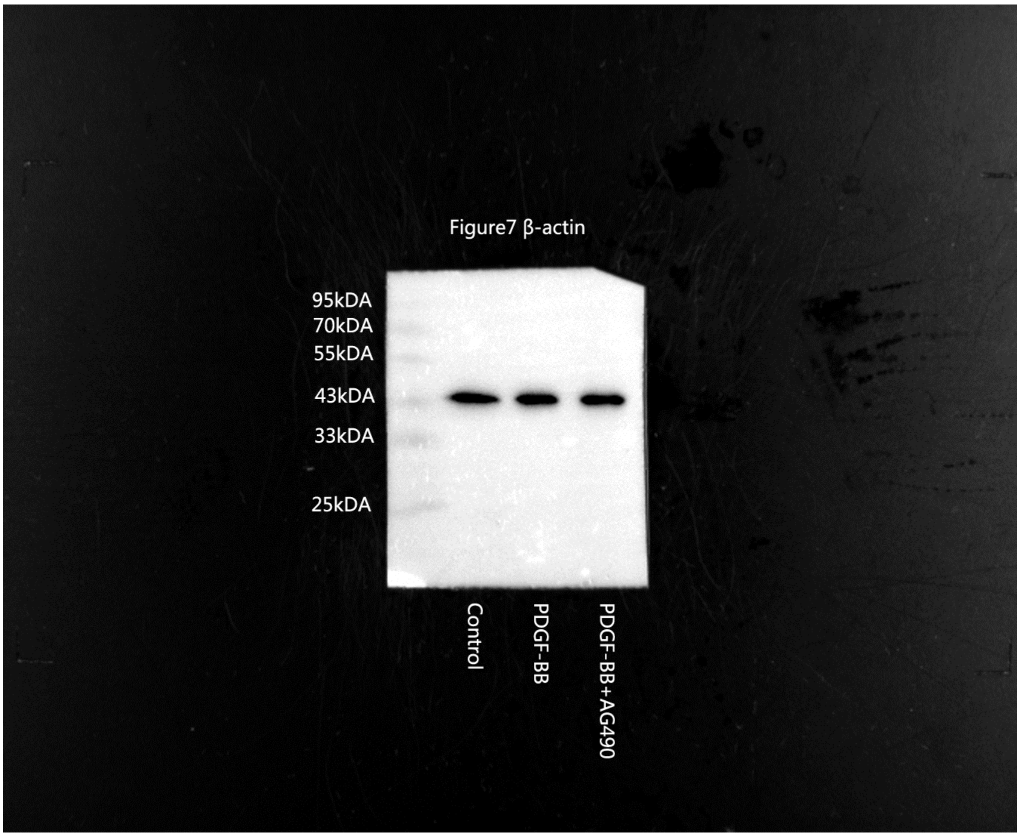

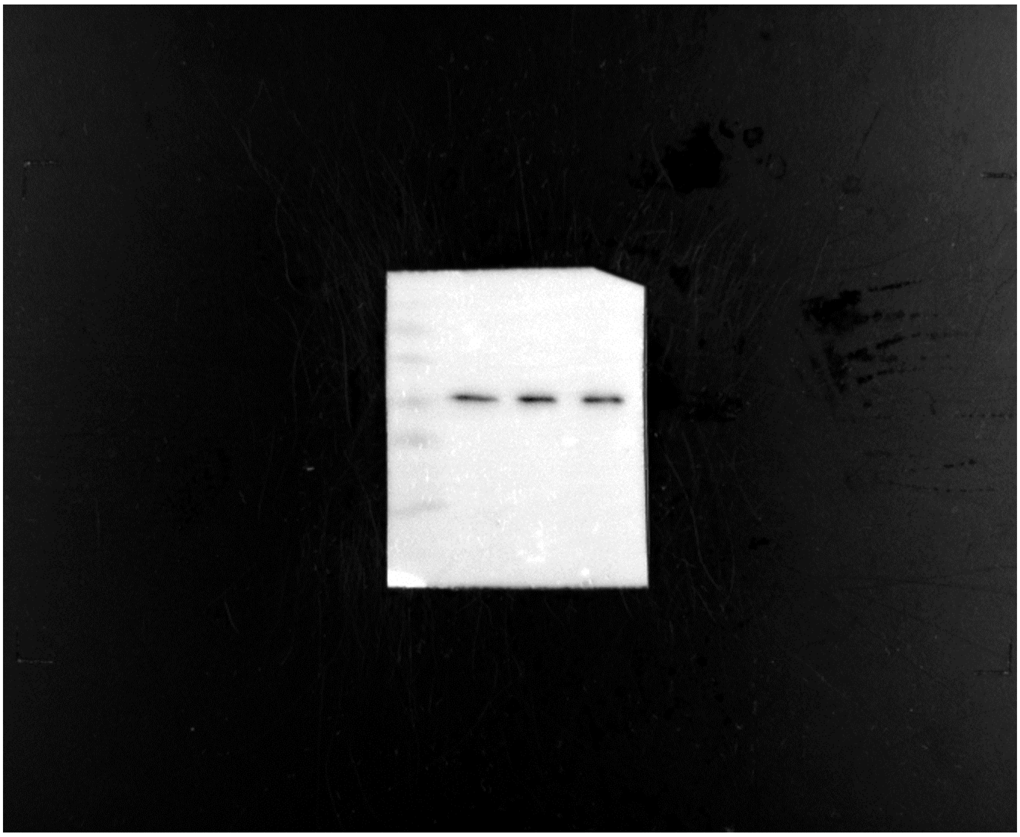

Supplement: Supplementary file 3 — Supplementary Information 3. [file 41598_2024_66482_MOESM3_ESM.pdf]
